# Evaluation of Substituted *N*-Phenylpiperazine Analogs as D3 vs. D2 Dopamine Receptor Subtype Selective Ligands

**DOI:** 10.3390/molecules26113182

**Published:** 2021-05-26

**Authors:** Boeun Lee, Michelle Taylor, Suzy A. Griffin, Tamara McInnis, Nathalie Sumien, Robert H. Mach, Robert R. Luedtke

**Affiliations:** 1 Department of Radiology, Perelman School of Medicine, University of Pennsylvania, Philadelphia, PA 19104, USA; boeunlee@pennmedicine.upenn.edu (B.L.); rmach@pennmedicine.upenn.edu (R.H.M.); 2 Department of Pharmacology and Neuroscience, University of North Texas Health Science Center-Fort Worth, Fort Worth, TX 76107, USA; michelle.taylor@unthsc.edu (M.T.); suzy.griffin@unthsc.edu (S.A.G.); tamara.mcinnis@unthsc.edu (T.M.); nathalie.sumien@unthsc.edu (N.S.)

**Keywords:** D2-like dopamine receptors, D3 dopamine receptor subtype, G-protein coupled receptor (GPCR), dopamine receptor subtype selective ligands, bitopic ligands

## Abstract

*N-*phenylpiperazine analogs can bind selectively to the D3 versus the D2 dopamine receptor subtype despite the fact that these two D2-like dopamine receptor subtypes exhibit substantial amino acid sequence homology. The binding for a number of these receptor subtype selective compounds was found to be consistent with their ability to bind at the D3 dopamine receptor subtype in a bitopic manner. In this study, a series of the 3-thiophenephenyl and 4-thiazolylphenyl fluoride substituted *N*-phenylpiperazine analogs were evaluated. Compound **6a** was found to bind at the human D3 receptor with nanomolar affinity with substantial D3 vs. D2 binding selectivity (approximately 500-fold). Compound **6a** was also tested for activity in two in-vivo assays: (1) a hallucinogenic-dependent head twitch response inhibition assay using DBA/2J mice and (2) an L-dopa-dependent abnormal involuntary movement (AIM) inhibition assay using unilateral 6-hydroxydopamine lesioned (hemiparkinsonian) rats. Compound **6a** was found to be active in both assays. This compound could lead to a better understanding of how a bitopic D3 dopamine receptor selective ligand might lead to the development of pharmacotherapeutics for the treatment of levodopa-induced dyskinesia (LID) in patients with Parkinson’s disease.

## 1. Introduction

Dopamine is a neurotransmitter that controls a variety of physiological functions in both the periphery and in the central nervous system (CNS). In the periphery, dopamine receptors have been implicated in the modulation of renal sodium excretion, urinary output, inhibition of norepinephrine release, vasodilatory activity of blood vessels and the regulation of insulin secretion in pancreatic β-cells [1,2]. Dopamine receptor expression has also been reported to be expressed on regulatory T cells and antigen-presenting dendritic cells associated with the immune system [3,4]. Dopaminergic pathways in the brain have been implicated in the reward system, psychostimulant seeking behaviors, movement, memory and learning, sleep regulation, feeding, attention, olfaction, vision, hormonal regulation, and emotional tone [5].

There are five human dopamine receptor subtypes classified into two major categories: D1-like (D1 and D5) and D2-like (D2, D3 and D4) dopamine receptor subtypes. The D1-like and D2-like dopamine receptor classes have been categorized based upon similarities in genomic organization, amino acid sequence homology and pharmacological properties [6]. All five of the dopamine receptor subtypes are members of the G protein-coupled receptor (GPCR) protein superfamily. The two genes which encode for the D1 and D5 are both intronless genes, whereas the D2, D3 and D4 dopamine receptor genes contain exons and introns. The length, organization, and amino acid homology of the two D1-like dopamine receptors are similar, with the D1-like receptors having a shorter third intracellular loop and a longer carboxy terminus compared to D2-like dopamine receptor subtypes. The three-dimensional structures of all three D2-like dopamine receptors has been determined by x-ray diffraction [7,8,9]. The structure of the D2 dopamine receptor in complex with a G*i*-protein has recently been reported using cryo-electron microscopy [10]. The three-dimensional structure of the D1-like receptors has not been reported.

Agonist stimulation of the D1-like dopamine receptors has been shown to increase the production of cAMP, whereas D2-like receptor agonists inhibit forskolin-dependent stimulation of adenylyl cyclase [11,12]. Agonist binding at both the D1-like and D2-like dopamine receptors leads to β-arrestin recruitment [13,14]. Dopamine receptor agonists are used therapeutically to treat Parkinson’s disease and restless legs syndrome, but adverse side effects can include nausea, orthostatic hypotension, hallucinations and impulse control disorders. Chronic administration of dopaminergic agonists that are used of L-dopa for the treatment of Parkinson’s disease in humans can lead to L-dopa-induced dyskinesia (LIDs). In preclinical studies, selective blockade of the D3 dopamine receptor has been reported to reduce L-dopa dependent dyskinesia-like movements, without affecting the antiparkinsonian efficacy of L-dopa [15,16].

Despite having similar amino acid sequences and pharmacologic profiles, the D2 and D3 dopamine receptor subtypes exhibit differences in how they couple to signal transduction pathways. For example, agonist stimulation of the D2 receptor activates ERK via a G_iα_-dependent pathway, whereas D3 receptors activate ERK by a mechanism that is G_βγ_ dependent [17]. In addition, sigma-1 receptors have been reported to selectively interact with D2 dopamine receptors, but not with the D3 or D4 receptor subtypes [18]. Further, the S100B calcium binding protein has been reported to interact with the D2, but not the D3, dopamine receptor [19].

Since the orthosteric binding site of D2-like dopamine receptors is constructed by conserved amino acid residues in the transmembrane helical spanning regions, it is not surprising that many of the original D2 vs. D1 selective ligands bind nonselectively at the D2 and D3 dopamine receptor subtypes. Ligands that can simultaneously interact with two different regions (an orthosteric and secondary binding site (SBS) on a single GPCR have been described as bitopic ligands [20,21,22,23,24]. The ability of some *N*-phenylpiperazine benzamides to bind selectively at the D3 dopamine receptor subtype has been attributed to the ability of the *N*-phenylpiperazine moiety to occupy either the D2 or D3 dopamine orthosteric binding site, while the benzamide moiety interacts with an SBS that is unique to the D3 dopamine receptor subtype [21,24]. Consequently, a number of research groups have been able to develop D3 vs. D2 dopamine receptor selective ligands using a substituted *N*-phenylpiperazine benzamide template to identify ligands that bind selectively and with high affinity at the D3 dopamine receptor subtype [25,26,27,28].

Previously we reported how incorporating a 4-(thiophen-3-yl)benzamide could lead to compounds that bind to the D3 receptor subtype with high affinity and with D3 vs. D2 receptor binding selectivity. Maintaining the thiophene moiety while varying the composition or position of substituents on the phenyl ring of the *N*-phenylpiperazine moiety leads to a panel of compounds with varying D2/D3 dopamine receptor subtype affinity and binding selectivity [22,23]. In this communication we explored the effect of varying the benzamide portion of our compounds while maintaining the fluorinated *N*-phenylpiperazine portion. The result of this study provides further insight into how structural variation in the dopaminergic ligands might impact ligand binding affinity and binding selectivity of *N*-phenylpiperazine benzamide at human D2 and D3 dopamine receptors.

## 2. Results

### 2.1. Chemistry

**Figure 1:**
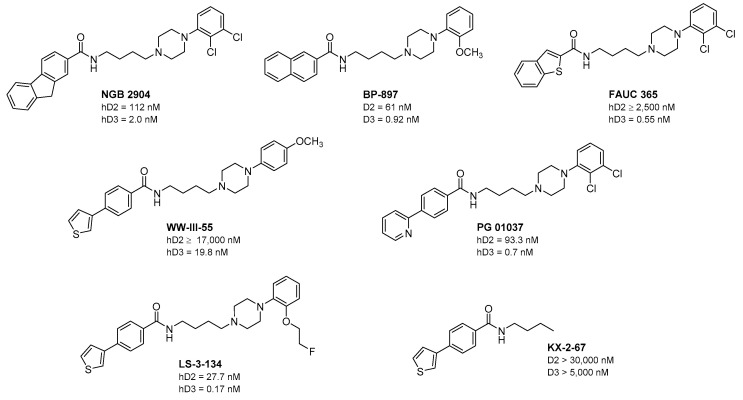
Chemical structures and binding data of a variety of arylamide phenylpiperazine D3 dopamine receptor selective compounds are shown. **KX-2-67** binds with low affinity to the secondary binding site of the D3 dopamine receptor [19,20,26,27,30].

The structure and binding affinities (K_i_ values) for several previously published *N*-phenylpiperazines at the human D2 and D3 dopamine receptor are shown in Figure 1. These compounds exhibit varying binding affinities at the D3 dopamine receptor and varying D3 vs. D2 binding selectivity [29,30,31,32,33]. Compound **LS-3-134** exhibits high affinity binding at the D3 receptor (K_i_ value of approximately 0.2 nM) with >150-fold D3 vs. D2 dopamine receptor binding selectivity, whereas **WW-III-55** exhibits a higher level of D3 vs. D2 dopamine receptor binding selectivity (>800-fold) but with lower binding affinity at the D3 dopamine receptor (K_i_ values approximately 20 nM). It is noteworthy that **LS-3-134** and **WW-III-55** both contain an *N*-phenylpiperazine and a 4-(thiophen-3-yl)benzamide moiety, while **KX-2-67** binds with low affinity predominantly to the SBS of the D2 and D3 dopamine receptor [21,22].

**Scheme 1:**
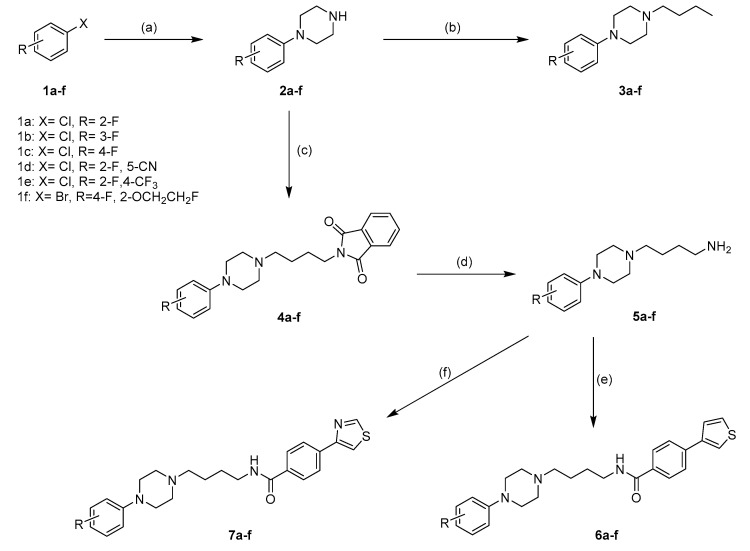
Synthesis of Thiophenephenyl and Thiazolylphenyl Substituted Phenylpiperazine Analogs. Reagents and conditions: (a) Aryl halide (**1a**–**f**), piperazine, NaOtBu, Pd_2_(dba)_3_, RuPhos, dioxane, 100 °C, 1 h; (b) 1-bromobutane, K_2_CO_3_, acetone, rt, overnight; (c) *N*-(4-bromobutyl)phthalimide, Et_3_N, acetonitrile, rt, overnight; (d) hydrazine hydrate, ethanol, 80 °C, 2 h; (e) 4-(thien-3-yl)benzoic acid, EDC, HOBt hydrate, CH_2_Cl_2_, rt, 1 h; (f) 4-(1,3-Thiazol-4-yl)benzoic acid, EDC, HOBt hydrate, CH_2_Cl_2_, rt, 1 h.

The synthesis strategy for the compounds described in this communication is outlined in Scheme 1. Briefly, the orthosteric *N*-phenylpiperazine moieties **2a**–**f** were afforded by a one-pot Pd-catalyzed Buchwald-Hartwig amination reaction of six different aryl halides with piperazine [34]. The *N*-alkylation of aryl piperazines **2a**–**f** with 1-bromobutane in acetone using potassium carbonate as a base provided fragments of D3 dopamine receptor selective compounds, **3a**–**f**. Butyl amine compounds **5a**–**f**, which were used for conjugation with substituted benzoic acids, were synthesized using a Gabriel amine synthesis reaction. *N*-arylpiperazine compounds **2a**–**f** were reacted with *N*-(4-bromobutyl)phthalimide in acetonitrile to give butyl phthalimide compounds **4a**–**f** and the hydrazinolysis of these compounds gave the corresponding amines **5a**–**f** in high yields (70–95%). The free amines **5a**–**f** were coupled with 4-(thien-3-yl)benzoic acid or 4-(1,3-thiazol-4-yl)benzoic acid using 1-ethyl-3-(3-dimethylaminopropyl)carbodiimide (EDC) and 1-hydroxybenzotriazole (HOBt) hydrate in dichloromethane to give the targeted benzamide compounds **6a**–**f** (52–88% yields) and **7a**–**f** (64–84% yields). The final products were converted to the corresponding hydrochloride salts. Detailed synthesis procedure and chemical analysis of synthesized compounds (^1^H, ^13^C NMR, and Mass) were reported in Supplementary Material.

### 2.2. Pharmacological Studies

#### 2.2.1. D2-Like Dopamine Receptors

Competitive radioligand binding techniques were used to determine the K_i_ values of ligands at human D2 and D3 dopamine receptors expressed in stably transfected HEK cells (Table 1). As anticipated, compounds **3a**–**f** exhibited the lowest binding affinities for the human D2 (K_i_ values = 349–7522 nM) and D3 (K_i_ values = 96–1413 nM) dopamine receptor subtypes. These compounds bound essentially nonselectively at the D2 and D3 dopamine receptor subtypes (binding selectivity ratio = 1.0–7.5-fold). The 4-thiophene-3-yl-benzamide *N*-phenylpiperazines (**6a**–**f**) and corresponding 4-thiazolyl-4-ylbenzamide *N*-piperazine analogs (**7a**–**f**) exhibited a similar range of binding affinities at the D3 dopamine receptor (K_i_ = 1.4–43 nM and 2.5–31 nM, respectively) and a similar range of D3 vs. D2 receptor binding selectivity (67–1831-fold vs. 73–1390-fold, respectively). Representative examples of competitive radioligand binding curves of compound **6a**–**c** and **7a**–**c** at human D2 and D3 dopamine receptors are shown in Supplementary Material.

A comparison of the binding affinity and binding selectivity of the *N*-phenylpiperazine orthosteric ligands (compounds **3a**–**f**) with the longer thiophene-containing (compounds **6a**–**f**) and thiazole-containing (compounds **7a**–**f**) analogs further suggest that the benzamide *N*-phenylpiperazine class of compound likely engage the D3 dopamine receptor, but not the D2 dopamine receptor, in a bitopic binding mode, leading to enhanced affinity at the D3 receptor subtype and consequently increased D3 vs. D2 binding selectivity. In general, the thiophene analogs (compounds **6a**–**f**) bound with slightly higher affinity at D3 dopamine receptors and/or with slightly greater D3 vs D2 binding selectivity than the corresponding thiazole analogs (compounds **7a**–**f**).

#### 2.2.2. 5-HT1A Receptor Binding

Because the 5-HT1A receptor is a commonly observed off-site binding site for benzamide *N*-phenylpiperazine ligands, compounds were simultaneously screened for binding of our compounds at the human 5-HT1A receptor expressed in CHO-K1 cells, using the radioligand [^3^H]-8-hydroxy-DPAT (8-OH-DPAT) (Table 2). Based upon the analysis of the displacement assay shown in Table 2, the orthosteric compounds **3c**–**f** appeared to bind with low affinity at human 5-HT1A receptors. However, at a concentration of 91 nM compounds **3a** and **3b** appeared to be able to partially displace the 5-HT1A selective radioligand. Compounds **6b, 7a, 7b** and **7f** displaced tritiated 8-OH-DPAT binding in a manner that suggested substantial affinity for the 5-HT1A receptor.

A comparison of the effect of compounds **3a, 6a** and **7a** on [^3^H]-8-hydroxy-DPAT binding was conducted. Based upon the radioligand displacement assessment the orthosteric ligand (compound **3a)** exhibited moderate affinity at 5-HT1A receptors. The thiazole analog of compound **3a** (compound **7a**) exhibited increased affinity at 5-HT1A, while the thiophene analog (compound **6a**) appeared to exhibit decreased binding affinity at the 5-HT1A receptor compared to compound **3a**.

**Figure 2:**
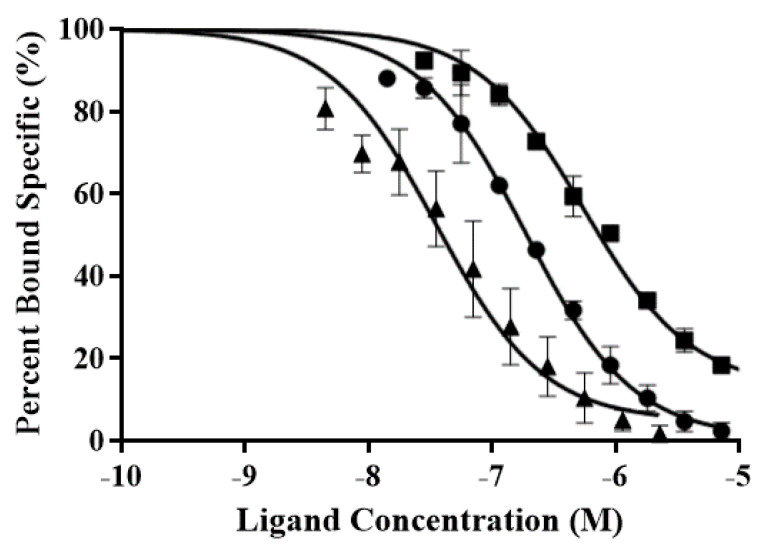
Competitive Radioligand Binding Assay for the Inhibition of [^3^H]-8-hydroxy-DPAT with 2-fluorophenyl Compounds at 5-HT1A Receptors**.** 2-Fluorophenyl piperazine compounds **3a** (–●–), **6a** (–■–), and **7a** (–▲–) were evaluated for their affinity at 5-HT1A receptor using competitive radioligand binding techniques. K_i_ values were determined by at least three experiments. Compound **7a** exhibited the highest affinity for the 5-HT1A receptor (K_i_ = 14.3 ± 7.1 nM) while compound **6a** had the lowest affinity for the receptor (K_i_ = 199 ± 34.3 nM). The orthosteric fragment, compound **3a**, exhibited an intermediate affinity for 5-HT1A receptors (K_i_ = 67.8 ± 4.6 nM). This rank-order of affinity is consistent with the results obtained in affinity displacement screen shown in Table 2.

Compound **6a** bound at the D3 dopamine receptor with high affinity (K_i_ value = 1.4 ± 0.21 nM) and exhibited >400-fold D3 vs. D2 receptor binding selectivity, while exhibiting a low level of radioligand displacement at 5-HT1A receptors, suggesting that it bound with low affinity at the 5-HT1A receptor binding site. To verify the low affinity binding of compound **6a** at the 5-HT1A receptor, a dose dependent competitive radioligand binding curve was performed. Compound **6a** was found to bind to the 5-HT1A receptor with a Ki value of 199 nM (Figure 2), which is approximately 140-fold lower than its affinity at the D3 dopamine receptor. The rank order of affinity of these analogs at the 5-HT1A receptor was found to be **7a** > **3a** > **6a** (Figure 2).

#### 2.2.3. Off-Target Binding Assessment

Further evaluation of the off-target binding of compound **6a** was provided by the Psychoactive Drug Screening Program (PDSP) for a range of receptors including serotonin receptor subtypes, α-adrenergic receptor subtypes, β-adrenergic receptor subtypes, histaminergic receptor subtypes, muscarinic receptor subtypes, opioid receptor subtypes and the two sigma receptor subtypes. The PDSP evaluation confirmed the high affinity binding of compound **6a** at the D3 dopamine receptor (0.2 nM) and the low affinity binding at the D1, D2, D4 (>1500 nM) and D5 dopamine receptor subtypes, as well as the dopamine transporter (DAT) (>190 nM), the norepinephrine transporter (NET) (>650 nM) and the serotonin transporter (SERT) (>750 nM). The highest off-site binding affinity was observed for the several of the alpha-adrenergic receptor subtypes (α_1_a, 9.8 nM; α_2_a, 15.2 nM; α_1_d, 16.6 nM; α_2_c, 27.1 nM), with lower affinity at the α_2_b (>100 nM) and α_1_b(>100 nM) receptor subtypes. Off-site binding was observed for three of the serotonin receptor subtypes (5-HT1A, 27 nM; 5-HT2B, 36.3 nM; 5-HT7A, 73.6 nM). Off-site binding affinity was higher at the sigma-2 receptor (33.1 nM) compared to the sigma-1 receptor (>175 nM).

#### 2.2.4. Ligand Efficacy Analysis

**Figure 3:**
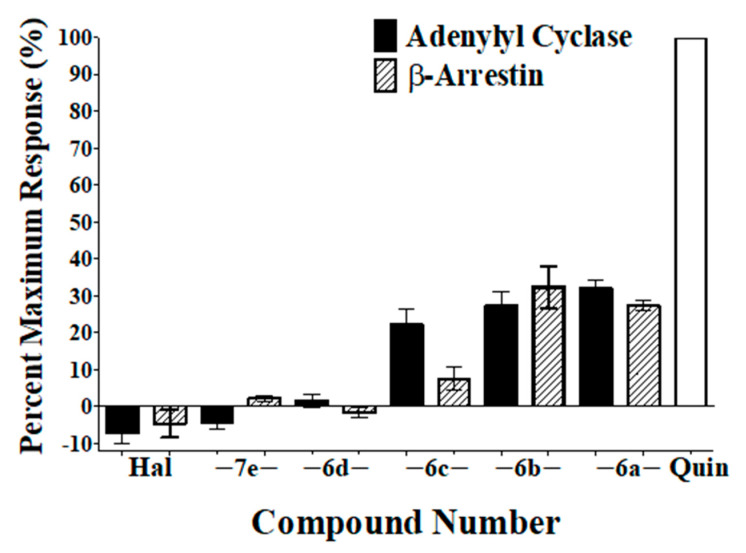
Comparison of the Efficacy of Phenylthiophene Analogs for Adenylyl Cyclase Inhibition and β-Arrestin Binding at Human D3 Dopamine Receptors**.** A comparison of the efficacy of compounds **6a**–**d** and **7e** for the inhibition of adenylyl cyclase and the binding of β-arrestin is shown. Values were normalized to reference full agonist quinpirole (10,000 nM). The concentration of the test compound was equal to 10× the K_i_ value as determined from competitive radioligand binding studies, therefore 90% of the binding sites were occupied.

Compounds exhibiting high affinity binding at the D3 dopamine receptor were subsequently evaluated for efficacy at the D3 dopamine receptor subtype using both a forskolin-dependent inhibition of adenylyl cyclase assay and a β-arrestin binding assay (Figure 3). Quinpirole was used as a reference prototypic full agonist and haloperidol was used as a reference prototypic antagonist. Compounds **7e** and **6d** were found to be antagonists in both assays. Compound **6c** was found to be functionally selective because it was a weak partial agonist for the adenylyl cyclase assay while being a very weak partial agonist/antagonist in the β-arrestin binding assay. Compounds **6a** and **6b** were found to be weak partial agonists in both the adenylyl cyclase inhibition assay and the β-arrestin binding assay.

### 2.3. In-Vivo Analysis of Compound **6a**

Based upon the data indicating that compound **6a** bound with high affinity to the human D3 dopamine receptor and with low affinity at both the human D2 dopamine receptor subtype (Table 1) and the 5-HT1A receptor (Table 2 and Figure 2), two in-vivo assays were performed to determine if compound **6a** was capable of in-vivo engagement of the D3 receptor in the brain. While we do appreciate the potential that possible in-vivo differences in pharmacological responses for the same drug in experimental animals and humans of sex differences, these initial in-vivo studies were primarily conduced to assess the ability of compound **6a** to cross the blood brain barrier (BBB) and engage the pharmacologically relevant target. Further sex differences in the pharmacological response of animals to compound 6a will be conduction as further in-vivo studies are necessitated.

**Figure 4:**
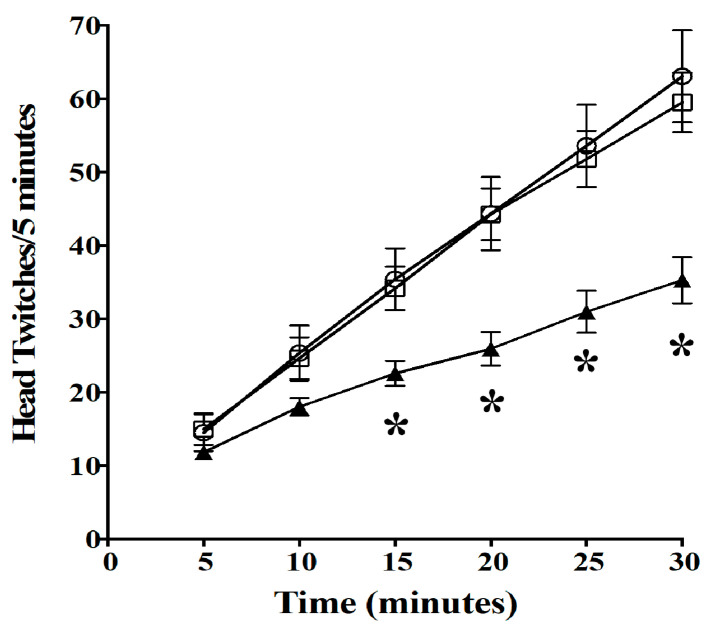
Inhibition of the Murine DOI-Dependent Head Twitch Response by Compound **6a.** The inhibition of the DOI-dependent Head Twitch Response (HTR) by compound **6a** as a function of time (minutes) is shown. Male DBA/2J mice were dosed with 5 mg/kg of DOI in the presence of 10 mg/kg of compound **6a**. The vehicle control studies were performed either one week before (–□–), or one week after (–○–) the compound **6a** (–▲–) was evaluated. The data shown is the mean number of head twitches ± S.E.M. averaged by two observers cumulatively at 5 min. Intervals for n = 7 mice. * A *p* < 0.05 was observed compared to both controls at times 15–30 min (one-way ANOVAs revealed main effects of Groups (F_2,18_ ranging from 5.1 to 10.4 with *p* < 0.019) and were followed by post-hoc analyses).

**Figure 5:**
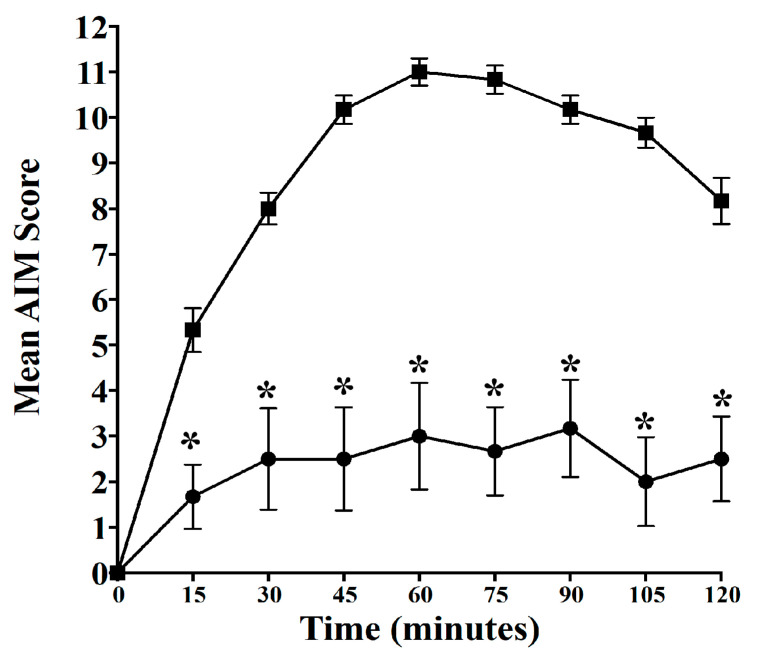
The Effect of Compound 6a on Abnormal Involuntary Movement (AIM) Scores in Hemiparkinsonian Rats**.** Male hemiparkinsonian rats were purchased from Charles Rivers Laboratories. After a 21 daily intraperitoneal injection regimen of 8 mg/kg of L-dopa and benserazide (each) the animals were administered L-dopa/benserazide (8 mg/kg each) either with vehicle (10% DMSO in sterile water) or compound **6a** at a dose of 10 mg/kg. Animals were scored on a scale from 0 to 4 for a) dystonic torsion of the trunk and neck contralateral to the lesion, b) dystonic movements of the forelimb contralateral to the lesion, c) orolingual movements with protrusion of the tongue and d) increased locomotion activity. Average AIM scores ± S.E.M. is shown for n = 6 animals in the presence (–●–) or absence (–■–) of compound **6a**. * A *p* < 0.05 value compared to vehicle control was observed for time points 15–120 min (one-way ANOVAs revealed main effects of Groups (F_1,10_ ranging from 11 to 38.4 with *p* < 0.009).

First, we have previously published that D3 receptor selective compounds could attenuate a hallucinogen-dependent head twitch response (HTR) in male DBA/2J mice [20,33]. Figure 4 indicates that at a dose of 10 mg/kg compound **6a** attenuated the number of head twitches induced by 5 mg/kg of the hallucinogen 2,5-dimethoxy-4-iodoamphetamine (DOI) over a 30 min time period. A two-way ANOVA with repeated measures revealed a main effect of Group (F_2,18_ = 6.49; *p* = 0.008), a main effect of Time (F_5,90_ = 348.16; *p* < 0.001) and an interaction between Time and Group (F_10,90_ = 14.92; *p* < 0.001). Second, we previously reported that D3 vs. D2 dopamine receptor subtype selective ligands could attenuate L-dopa dependent abnormal involuntary movement (AIM) scores in unilaterally lesioned (hemiparkinsonian) male rats, which is a model for L-dopa-Induced Dyskinesia (LID) [35,36]. Figure 5 indicates that at a dose of 10 mg/kg compound **6a** could attenuate the AIM scores induced by 8 mg/kg of L-dopa and benserazide (each) in unilaterally lesioned rats. A two-way ANOVA with repeated measures revealed a main effect of Group (F_1,10_ = 26.98; *p* < 0.001), a main effect of Time (F_7,70_ = 15.61; *p* < 0.001) and an interaction between Time and Group (F_7,70_ = 7.44; *p* < 0.001).

## 3. Discussion

This communication describes part of our ongoing studies on the development of dopamine receptor selective ligands in vivo to dissect the role of the D2 and D3 dopamine receptor subtypes in the rewarding and motivational aspects of psychostimulant abuse [35,37,38,39], the treatment of neurological and neurodegenerative disorders [36,40] and for the development of selective imaging agents [41].

We previously reported that structural variations in the aryl portion of 4-(thiophen-3-yl)benzamides play an important role in D3 dopamine receptor affinity, D3 vs. D2 receptor binding selectivity and D3 dopamine receptor functional selectivity (biased agonism) [20,21,22,30,35,42]. The structural templates for these studies included **LS-3-134** and **WW-III-55** (Figure 1). Both compounds possess a common 4-(thiophen-3-yl)benzamide with a saturated four-carbon chain adjacent to a substituted phenylpiperazine moiety. **LS-3-134** was found to bind at the human D3 dopamine receptor with high affinity (K_i_ = 0.17 nM) and demonstrated >150-fold D3 vs. D2 dopamine receptor binding selectivity [30]. **LS-3-134** was found to be a partial agonist at the D3 dopamine receptor (35% of maximum efficacy) using an adenylyl cyclase inhibition assay. While **WW-III-55** exhibits only moderate binding affinity (approximate K_i_ of 20 nM) at D3 dopamine receptors, this compound binds with high (>800-fold) D3 vs. D2 dopamine receptor binding selectivity. Using an adenylyl cyclase assay, **WW-III-55** was found to be a strong partial agonist (67.6 ± 12.5% of maximum efficacy) while **LS-3-134** was found to be a weak partial agonist (34.4 ± 1.7%). Therefore, we projected that structurally related analogs of **WW-III-55** and **LS-3-134** might be identified that could exhibit high affinity binding at the D3 dopamine receptor, substantial D3 vs. D2 receptor binding selectivity and varying efficacy for G-protein and β-arrestin mediated signaling.

This expectation was verified when we examine the binding properties of a panel of structural analogs of **WW-III-55** and **LS-3-134**, where the 4-(thiophen-3-yl)benzamide structure, which we project interacts with the SBS, was invariant while substituents on the phenyl moiety of the phenylpiperazine (the structure binding to the orthosteric binding site) were varied. This synthetic strategy led to the identification of several compounds that exhibited subnanomolar affinity (Ki values < 1.0 nM) at the human D3 dopamine receptor subtype with high D3 vs. D2 dopamine receptor binding selectivity (≥1000-fold) [22]. In the context of a dynamic ensemble theory of GPCR structure and activation [43,44,45], this observation suggests that each of the 4-(thiophen-3-yl)benzamide analogs used in that study interacts with the D3 dopamine receptor protein in a slightly different manner (depending on the position and chemical properties of the substituent), stabilizing a unique ensemble of receptor structures resulting in a collection of ligand-dependent structural variations in the D3 dopamine receptor energy landscape [42,43,44,45].

In this communication our initial goal was first to characterize a number of synthetic analogs in which the 4-(thiophen-3-yl)benzamide group and the adjacent saturated four carbon chain was invariant, while the substitution pattern on the phenyl ring of the phenylpiperazine moiety substituted at the 2nd, 3rd and 4th position with a fluorine. Once we established that the 2nd position was optimal for a fluoride substitution (compounds **3a** and **6a**), our second goal was to attempt to further optimize ligand binding affinity by exploring additional substitutions on the phenyl ring of the *N*-phenylpiperazine moiety (compounds **6d**–**f**). Compound 6a remained our best candidate, with the highest affinity at the D3 receptor subtype (Ki value = 1.4 nM), while it exhibited substantial D3 vs. D2 receptor binding selectivity (>450-fold). Our third synthetic strategy was to substitute the thiophene ring (**6a**–**f**) with a thiazole ring (**7a**–**f**). In general, this structural alteration led to a decrease in D3 vs. D2 receptor binding selectivity.

Using the aforementioned strategy, we were able to identify a number of ligands which exhibited high affinity binding at the D3 dopamine receptor or substantial D3 vs. D2 dopamine receptor binding selectivity. However, compound 6a remained of particular interest because it bound at the D3 dopamine receptor with high affinity (K_i_ value = 1.4 nM) and exhibited substantial D3 vs. D2 binding selectivity (>400-fold).

A direct comparison of the binding affinities for compounds **3a**–**f** and **6a**–**f** suggests that compound **6a**–**f** likely bound at the D3 dopamine receptor subtype, but not the D2 dopamine receptor, in a bitopic mode. This observation was similar to what we previously reported [23]. While the identification of a ligand that can interact with a dualistic ligand binding mode may not be applicable for the development of receptor subtype selective ligands for every GPCR subfamily, it appears not to be unique to the dopamine receptor subtypes. Bitopic ligands have also been reported for other GPCRs, including the muscarinic and angiotensin II receptors [46,47,48]. Furthermore, binding data provided by NIMH Psychoactive Drug Screening Program (PSDP) indicated that compound 6a had reduced affinity at the 5-HT1A receptor, which is a common off-site binding component that has limited the development of in-vivo D3 dopamine receptor selective imaging agents. However, this binding data indicated that several alpha-adrenergic receptor subtypes bound compound **6a**.

The studies in this communication also demonstrated that after intraperitoneal injection compound **6a** was capable of engaging CNS D3 dopamine receptors in vivo leading to a significant decrease in the DOI-dependent head twitch response in mice and AIM scores in dyskinetic hemiparkinsonian rats. These observations suggest that compound **6a** was capable of traversing the blood-brain barrier in a manner such that a therapeutic concentration could be achieved. We anticipate that studies such as those reported in this communication could lead to a better understanding of how the development of bitopic ligands might be designed and used to selectively target structurally similar GPCR receptor subtypes in vivo.

## 4. Materials and Methods

### 4.1. Chemistry

All reagents and solvents for synthesis were purchased and used without further purification. Structures of synthesized chemicals were identified using ^1^H and ^13^C nuclear magnetic resonance (NMR) spectra, and mass spectroscopy. ^1^H and ^13^C NMR were recorded in δ units relative to deuterated solvent (CDCl_3_) as an internal reference by Bruker DMX 500 MHz NMR instrument (Bruker, Billerica, MA, USA). ^1^H chemical shifts are reported in parts per million (ppm) and measured relative to tetramethylsilane (TMS). Mass spectra were acquired using a 2695 Alliance LC/MS (Milford, MA, USA). Purification of synthesized chemicals was conducted on Biotage Isolera One (Biotage, Salem, NH, USA) with a dual-wavelength UV-VIS detector. A detailed description of the synthesis and characterization of the compounds described in this paper can be found in the Supporting Information.

### 4.2. D2 and D3 Dopamine Receptor Competitive Radioligand Binding Assays

For these competitive binding studies, transfected HEK293 cell homogenates were suspended in homogenization buffer and incubated with radioligand [^125^I]IABN, in the presence or absence of inhibitor at 37 °C for 60 min (total volume = 150 μL), as previously described [49]. The final radioligand concentration was approximately equal to the K_d_ value for the binding of the radioligand. Nonspecific binding was defined as the binding of the radioligand in the presence of 25 µM (+)-butaclamol. For each competition curve, triplicates were performed using two concentrations of inhibitor per decade over five orders of magnitude. Binding was terminated by the addition of cold wash buffer (10 mM Tris–HCl/150 mM NaCl, pH = 7.5) and filtration over a glass-fiber filter (Pall A/B filters, #66198). A Packard Cobra Gamma Counter (PerkinElmer, Waltham, MA, USA) was used to measure the radioactivity of [^125^I]IABN.

The competition curves were modeled for a single binding site using

Bs = Bo − ((Bo + L)/(IC_50_ + L))

where Bs is the amount of ligand bound to receptor and Bo is the amount of ligand bound to receptor in the absence of competitive inhibitor. L is the concentration of the competitive inhibitor. The IC_50_ value is the concentration of competitive inhibitor that inhibits 50% of the total specific binding. IC_50_ values were determined using non-linear regression analysis with TableCurve 2D v 5.01 (Jandel, SYSTAT, Systat Software, Inc., San Jose, CA, USA). The values for Bs and Bo were constrained using experimentally derived values. The IC_50_ values were converted to equilibrium dissociation constants (K_i_) [50,51]. Mean K_i_ values ± S.E.M. are reported for at least three independent experiments.

### 4.3. 5-HT1A Receptor Binding Assays

10 μg of membranes from CHO-K1 cells expressing the human 5-HT1A receptor were suspended in 50 mM Tris-HCl, 10 mM MgSO_4_, 0.5 mM EDTA, and 0.1% (*w*/*v*) ascorbic acid, pH 7.4 and test ligands (91 nM for single point radioligand displacement assay and 1 nM to 1 μM for full competition assay) were incubated with 0.25 nM [^3^H]8-hydroxy-DPAT (PerkinElmer, Boston, MA, USA) for 60 min at room temperature. The nonspecific binding was determined using 10 μM metergoline (Sigma, St. Louis, MO, USA). After incubation, the bound ligands were filtered using an M-24 Brandel filtration system (Brandel, Gaithersburg, MD, USA), collected on glass fiber papers (Whatman grade 934-AH, GE Healthcare Bio-Sciences, Pittsburgh, PA, USA), and the radioactivity was quantitated using a MicroBeta2 Microplate scintillation counter 2450 (PerkinElmer, Boston, MA, USA).

### 4.4. Ligand Efficacy Analysis

#### 4.4.1. Cyclase Inhibition Assay

The forskolin-dependent adenylyl cyclase inhibition assay was performed using a cAMP-Glo Assay kit (Promega). For this assay cAMP stimulates protein kinase A (PKA) activity, which leads to a decrease in ATP levels and a decrease in the luciferase-coupled production of light. This is a 96 well plate assay using HEK cells stably transfected to express the human D3 dopamine receptor (10,000 cells/well/0.1 mL complete media). After removing media and rinsing with phosphate buffered saline, cells were incubated with 20 μL of forskolin (6000 nM) and test compound or vehicle. The dose of test compound was equal to 10x the K_i_ value obtained from our competitive radioligand binding studies, such that 90% of the binding sites are occupied. After cells, test compound (or vehicle) and forskolin (or vehicle) were incubated for 15 min, cells were lysed using 20 μL lysis buffer (provided with the kit) and mixed for 15 min at room temperature. A cAMP Detection Solution (40 μL) was added to each well, gently mixed for 60 s and incubated at room temperature for 20 min. The Kinase-Glo Solution was added (40 μL) to terminate the PKA reaction. The reaction mixture was gently mixed for 60 s and then incubated at room temperature for 10 min. The remaining ATP was detected via a luciferase reaction. The plates were read using an Enspire Alpha 2390 Multilabel Reader/Luminometer. Haloperidol (200 nM) and quinpirole (10,000 nM) was used as a prototypic reference antagonist and full agonist, respectively.

#### 4.4.2. β-Arrestin Binding Assay

The β-arrestin binding assay was performed using a DiscoverX Pathhunter^®^ kit according to the manufacturer’s instructions. PathHunter^®^ eXpress β-Arrestin GPCR cells are engineered to co-express the ProLink™ (PK) tagged GPCR and the Enzyme Acceptor (EA) tagged β-Arrestin. Activation of the GPCR-PK induces β-Arrestin-EA recruitment, forcing complementation of the two β-galactosidase enzyme fragments (EA and PK). The resulting functional enzyme hydrolyzes substrate to generate a chemiluminescent signal. DRD3-U2OS cells were seeded according to kit instructions into white plastic 96-well plates (provided with the kit) 48 h prior to the assay in a volume of 100 μL/well using the Cell Plating Reagent (CPR) provided. Vehicle or test drug, diluted in CPR, was added to the designated wells and incubated for 90 min at 37 °C. Working detection solution (a component provided with the kit) was added to the wells and incubated for 1 h in the dark at room temperature. As discussed for the cyclase assay, an Enspire Alpha 2390 Multilabel Reader/Luminometer was used to detect the luminescent signal with haloperidol and quinpirole used as reference antagonist and full agonist, respectively.

### 4.5. In-Vivo Studies

The treatment of the animals and the experimental procedures were approved by the UNTHSC Institutional Animal Care and Use Committee (IACUC). Animal care and housing were in adherence with the conditions set forth in the “Guide for the Care and Use of Laboratory Animals” (Institute of Laboratory Animal Resources on Life Sciences, National Research Council, 1996).

#### 4.5.1. Head Twitch Response

Upon arrival DBA/2J mice (Jackson Labs) were allowed to acclimate for at least one week prior to testing. Mice were housed at ≤4 animals per cage with unlimited access to food and fresh water. The protocol for the HTR assessment has been previously reported [32]. Briefly, on the day of testing mice were weighed and placed individually in an open-ended Plexiglas cylinder (21 × 34 cm) with a clean paper towel on the floor in a dimly lighted room. Animals were allowed to habituate to the cylinder for 10 min prior to the intraperitoneal (i.p.) injection of vehicle (90% sterile deionized water and 10% dimethylsulfoxide (DMSO: Sigma) in sterile deionized water) or test drug (10 mg/kg). Five minutes later DOI (5 mg/kg) was administered, and the mouse was immediately returned to the cylinder.

The mice exhibited various responses to DOI administration including a vertical jerking movement. However, a head twitch was defined as a rapid movement of the head, without the involvement of the front paws during grooming. We estimate that the HTR lasted approximately 0.6 s. At least two observers counted the number of head twitches by visual examination and the number of head twitches was recorded in 5-min cumulative intervals. The data points are presented as the mean values obtained by two observers.

The effect of Group was assessed using two-way analysis of variance (ANOVA) with Time as the repeated measure. The data was further analyzed at each time point with a one-way ANOVA with Group as the factor followed by planned individual comparisons between different groups using a single degree-of-freedom F test involving the error term from the overall ANOVA when the Time and Group interaction was significant. The alpha level was set at 0.05 and Systat 13 statistical package was used.

#### 4.5.2. AIMs Studies

The Abnormal Involuntary Movement (AIMs) studies were performed using male Sprague-Dawley rats that were unilaterally lesioned, using injections of 6-hydroxydopamine (6-OHDA) in the medial forebrain bundle (MFB) by the commercial vendor (Charles River Laboratories). Upon arrival to our vivarium, the lesioned rats were housed under a 12-h light: 12-h dark cycle. The animals were housed singly with unlimited access to food and water. 8 mg/kg L-dopa with 8 mg/kg benserazide was administered to each rat as a daily i.p. injection for 21 consecutive days to induce the development of dyskinesia-like movements. Benserazide was included because it is a peripherally-acting aromatic L-amino acid decarboxylase inhibitor which prevents the peripheral metabolism of L-dopa. The L-dopa/benserazide solutions were dissolved in water. Test drug (compound **6a**) was prepared using 90% sterile water with 10% DMSO.

8 mg/kg of L-dopa combined with 8 mg/kg benserazide was administered via intraperitoneal injection followed by either the vehicle control or test drug (compound **6a**, 10 mg/kg). Experiments were designed such that animals received test drug only once per week. Throughout the course of these studies each animal received a minimum of one dose of L-dopa/benserazide (8 mg/kg each) per week to maintain the involuntary movements.

AIMs ratings were performed as previously described [31,38,52]. The severity of the AIMs was quantified using lesioned rats that were observed individually in their home cages at 15 min intervals, starting 15 min after the injection of L-dopa for approximately 2 h. The AIMs were scored in four categories: (1) axial AIMs, including dystonic or choreiform torsion of the trunk and neck towards the side contralateral to the lesion; (2) limb AIMs, including jerky and/or dystonic movements of the forelimb contralateral to the lesion; (3) orolingual AIMs, including twitching of orofacial muscles with empty masticatory movements and protrusion of the tongue towards the side contralateral to the lesion and (4) locomotive AIMs, including increased locomotion with contralateral side bias. Each of the four categories were scored on a severity scale from 0 to 4, where 0 = absent, 1 = present for less than half of the observation time, 2 = present for more than half of the observation time, 3 = present all the time but suppressible by external stimuli and 4 = present all the time and not suppressible by external stimuli. For these experiments the external stimuli that was used was gentle tapping on the cage with a pencil.

The change in crossovers from controls was calculated for each test compound dosage group by subtracting the average number of crossovers of the respective vehicle group from the number of crossovers of individual subjects in the test compound dosage groups. The latter measure is graphed to illustrate the effects of the compounds on locomotion.

The effect of Group was assessed using two-way analysis of variance (ANOVA) with time as the repeated measure. The data was further analyzed at each time point with a one-way ANOVA with Group as the factor. The alpha level was set at 0.05 and Systat 13 statistical package was used.

## Figures and Tables

**Table 1:**
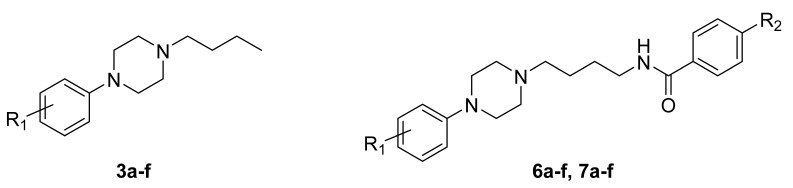
Binding Affinity and cLogP Values for Compounds.

#	Structure		*K*_i_ Values (nM) ^1^		D2:D3 Selectivity	cLogP ^2^
	R_1_	R_2_	D2 Receptor	D3 Receptor		
3a	2-F	−	384 ± 39.6	96.2 ± 16.9	4.0	4.06
3b	3-F		2516 ± 252	1003 ± 118	2.5	4.06
3c	4-F		3091 ± 269	1176 ± 240	2.6	4.06
3d	2-F, 5-CN		5431 ± 1059	726 ± 101	7.5	3.78
3e	2-F, 4-CF_3_		7522 ± 466	1413 ± 59.1	5.3	5.13
3f	2-OC_2_H_4_F, 4-F		349 ± 21.1	351 ± 42.1	1.0	4.31
6a	2-F	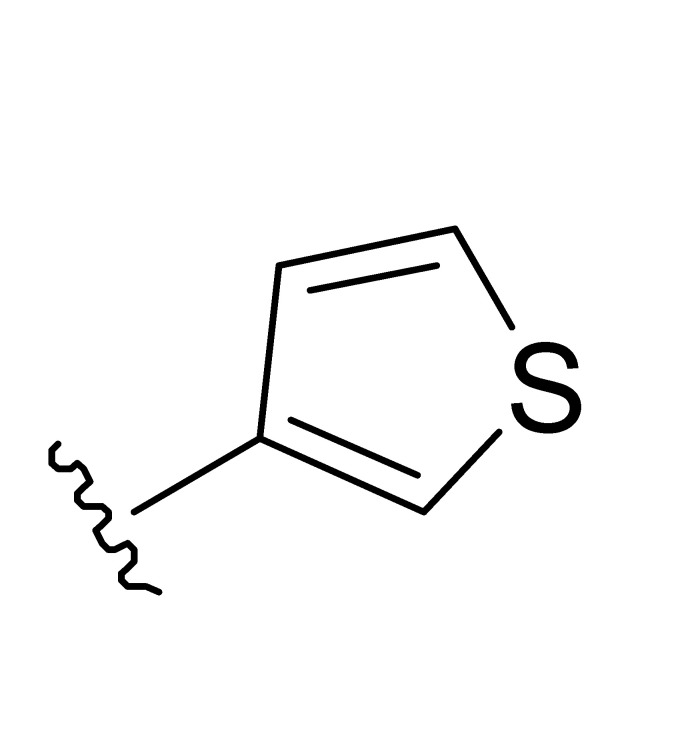	648 ± 100	1.4 ± 0.2	467	5.15
6b	3-F		2334 ± 485	7.9 ± 1.4	296	5.15
6c	4-F		7200 ± 215	12.4 ± 1.6	579	5.15
6d	2-F, 5-CN		4502 ± 565	15.5 ± 1.3	290	4.87
6e	2-F, 4-CF_3_		>75,000	43.3 ± 9.2	>1800	6.22
6f	2-OC_2_H_4_F, 4-F		411 ± 27.3	6.1 ± 0.2	67.4	5.40
7a	2-F	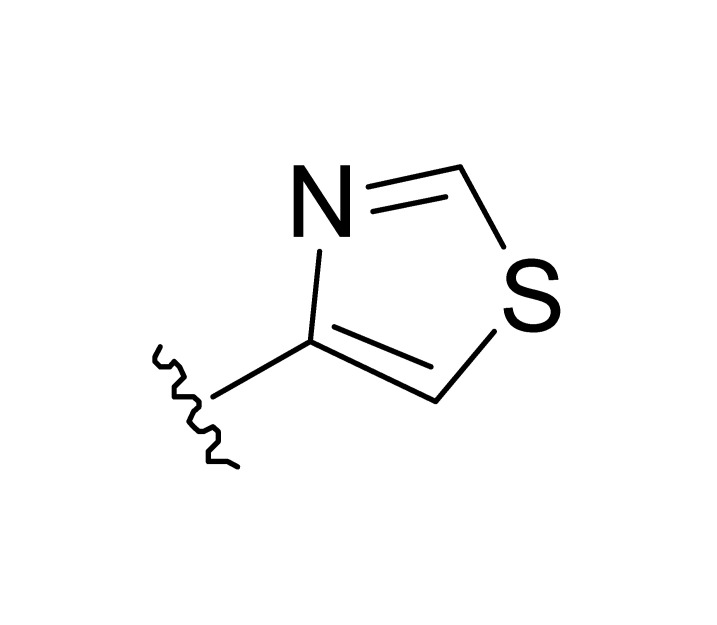	478 ± 71.0	2.5 ± 0.22	190	4.16
7b	3-F		3946 ± 146	24.0 ± 5.5	165	4.16
7c	4-F		4012 ± 694	28.6 ± 2.1	140	4.17
7d	2-F, 5-CN		7717 ± 1497	28.1 ± 0.8	274	3.89
7e	2-F, 4-CF_3_		>40,000	30.9 ± 2.6	1390	5.24
7f	2-OC_2_H_4_F, 4-F		349 ± 50.3	4.8 ± 0.28	72.9	4.42

The K_i_ values for the binding of test compound at human D2 and D3 dopamine receptors are shown. The D3 vs. D2 dopamine receptor binding selectivity and cLogP values of the test compound are also shown. ^1^ Mean ± S.E.M.; K_i_ values were determined by at least three experiments. ^2^ cLogP values were calculated using ChemDraw Professional 15.1.

**Table 2:**
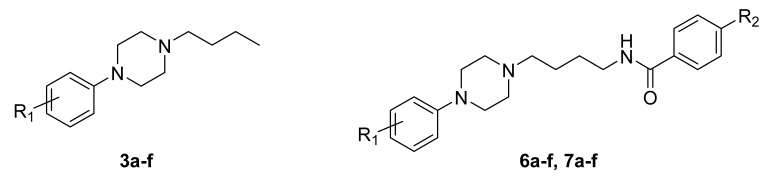
Screen for Binding Affinity at the 5-HT1A Receptor.

#	Structure		5-HT1A Receptor Inhibition (%)
	R_1_	R_2_	
3a	2-F	−	20.3 ± 16.2
3b	3-F		36.1 ± 2.1
3c	4-F		5.1 ± 11.2
3d	2-F, 5-CN		−18.0 ± 11.1
3e	2-F, 4-CF_3_		−11.6 ± 15.4
3f	2-OC_2_H_4_F, 4-F		1.7 ± 21.7
6a	2-F	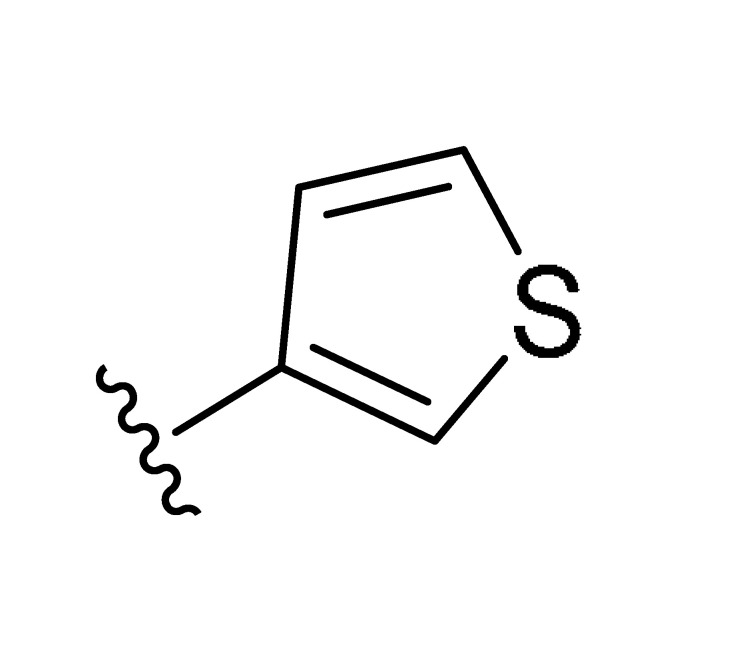	−3.3 ± 15.1
6b	3-F		74.7 ± 1.9
6c	4-F		−7.7 ± 23.5
6d	2-F, 5-CN		2.5 ± 3.5
6e	2-F, 4-CF_3_		6.1 ± 6.8
6f	2-OC_2_H_4_F, 4-F		16.9 ± 24.4
7a	2-F	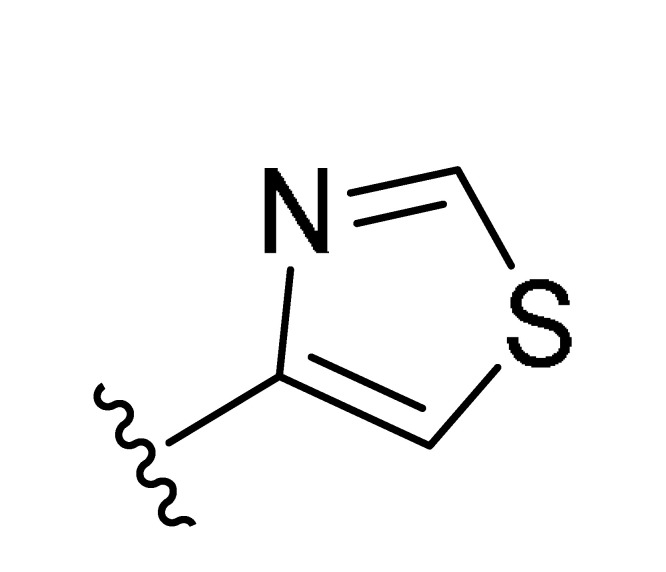	65.8 ± 5.0
7b	3-F		74.1 ± 5.3
7c	4-F		12.2 ± 3.1
7d	2-F, 5-CN		−5.6 ± 18.3
7e	2-F, 4-CF_3_		7.6 ± 6.5
7f	2-OC_2_H_4_F, 4-F		37.1 ± 24.4

Phenylpiperazine ligands **3a**–**f**, **6a**–**f**, and **7a**–**f** were screened for the binding activity of [^3^H]-8-hydroxy-DPAT at human 5-HT1A receptors expressed in CHO-K1 cells using a single point radioligand displacement assay. The radioligand displacement percentages were determined by at least three experiments using 91 nM concentration of test compound. Nonspecific binding was defined using 10 μM metergoline. Compounds **3a** and **3b** exhibited moderate displacement of the radioligand at the 5-HT1A receptor. Thiophenyl compound **6b** and thiazolyl compounds **7a**, **7b**, and **7f** exhibited a greater percentage of radioligand displacement. Data is presented as percent displacement of radioligand (n ≥ 3) ± S.E.M.

## Data Availability

The article contains complete data used to support the findings of this study.

## Supplementary Materials

The following are available online, procedure of synthesis; chemical analysis of synthesized compounds (^1^H, ^13^ C NMR, and Mass); Figure S1: representative examples of competitive radioligand binding studies for human D2 and D3 dopamine receptors (compound **6a**–**c** and **7a**–**c**).

## Sample Availability

Samples of the compounds described in this paper are available from the authors.
