# Supplementary material for: Evaluation of Substituted N-Phenylpiperazine Analogs as D3 vs. D2 Dopamine Receptor Subtype Selective Ligands
Source: Molecules. 2021 May 26;26(11):3182. doi: 10.3390/molecules26113182 (PMC8198181; doi:10.3390/molecules26113182)
Supplement: Supplementary file 1 [file molecules-26-03182-s001.zip › molecules-1219670-supplementary.pdf]

molecules

MDPI

Supporting Information

Evaluation of Substituted *N*-Phenylpiperazine Analogs as D3 vs. D2 Dopamine Receptor Subtype Selective Ligands

Boeun Lee [1], Michelle Taylor [2], Suzy A. Griffin [2], Tamara McInnis [2], Natalie Sumien [2], Robert H. Mach [1], and Robert R. Luedtke [2,*]

[1] Department of Radiology, Perelman School of Medicine, University of Pennsylvania, Philadelphia, PA 19104, USA; boeunlee@pennmedicine.upenn.edu (B.L.), rmach@pennmedicine.upenn.edu (R.H.M.)
[2] Department of Pharmacology and Neuroscience, University of North Texas Health Science Center-Fort Worth, Texas, TE 76107, USA; michelle.taylor@unthsc.edu (M.T.), suzy.griffin@unthsc.edu (S.A.G.), tamara.mcinnis@unthsc.edu (T.M.), nathalie.sumien@unthsc.edu (N.S.)
* Correspondence: robert.luedtke@unthsc.edu (R.R.L.)

1. Synthesis procedures and chemical analysis

General procedure for the synthesis of compound ***2a–f****.* A solution of aryl halide (1 equiv), piperazine (4 equiv), NaOtBu (1.5 equiv), $Pd_2(dba)_3$ (1–5 mol%), RuPhos (2–10 mol%) in dioxane was stirred at 100 °C for 1 h. After cooling to room temperature, the crude reaction mixture was filtered and concentrated under reduced pressure. The residue was purified by flash chromatography on silica gel by elution with a 0–10 % gradient of 7N ammonia solution in methanol and CH_2Cl_2.

*1-(2-Fluorophenyl)piperazine (**2a**).* Following the general procedure, **2a** (753 mg, 54.5%) was obtained as a yellow oil from 1-chloro-2-fluorobenzene: ^{1}H NMR ($CDCl_3$, 500 MHz) δ 1.84 (s, 1H) 3.04 (s, 8H), 6.90-6.96 (m, 2H), 6.98-7.06 (m, 2H); ^{13}C NMR ($CDCl_3$, 125 MHz) δ 156.3, 154.4, 139.9, 139.8, 124.2, 124.1, 122.4, 122.3, 118.7, 115.8, 115.7, 50.6, 45.3; LC-MS (ESI) *m/z:* 181.24 [M+H].

*1-(3-Fluorophenyl)piperazine (**2b**).* Following the general procedure, **2b** (874 mg, 51.9%) was obtained as a yellow oil from 1-chloro-3-fluorobenzene: ^{1}H NMR ($CDCl_3$, 500 MHz) δ 1.92 (s, 1H), 2.99 (t, *J* = 5.0 Hz, 4H), 3.12 (t, *J* = 5.0 Hz, 4H), 6.50 (td, *J* = 8.2, 2.4 Hz, 1H), 6.56 (dt, *J* = 8.6, 6.2 Hz, 1H), 6.64 (dd, *J* = 5.3, 2.2 Hz, 1H), 7.16 (q, *J* = 8.2 Hz, 1H); ^{13}C NMR ($CDCl_3$, 125 MHz) δ 164.7, 162.8, 153.3, 130.0, 111.0, 105.7, 105.6, 102.6, 102.4, 49.6, 45.8; LC-MS (ESI) *m/z:* 181.24 [M+H].

*1-(4-Fluorophenyl)piperazine (**2c**).* Following the general procedure, **2c** (560 mg, 81.1%) was obtained as a yellow oil from 1-chloro-2-fluorobenzene: ^{1}H NMR ($CDCl_3$, 500 MHz) δ 2.76 (s, 1H), 3.06-3.11 (m, 8H), 6.86-6.89 (m, 2H), 6.94-6.98 (m, 2H); ^{13}C NMR ($CDCl_3$, 125 MHz) δ 158.2, 156.3, 148.2, 118.1, 118.0, 115.6, 115.4, 50.9, 45.8; LC-MS (ESI) *m/z:* 181.24 [M+H].

*4-Fluoro-3-(piperazin-1-yl)benzonitrile (**2d**).* Following the general procedure, **2d** (397 mg, 50.0%) was obtained as a brown waxy solid from 3-chloro-4-fluorobenzonitrile: ^{1}H NMR ($CDCl_3$, 500 MHz) δ 1.83 (s, 1H), 2.98-3.27 (m, 8H), 6.83-7.48 (m, 3H); ^{13}C NMR ($CDCl_3$, 125 MHz) δ 158.9, 156.9, 141.1, 141.0, 133.4, 126.7, 126.6, 122.7, 122.6, 118.3, 117.4, 117.2, 114.2, 108.7, 108.6, 50.4, 45.4; LC-MS (ESI) *m/z:* 206.31 [M+H].

*1-(2-Fluoro-4-(trifluoromethyl)phenyl)piperazine (**2e**).* Following the general procedure, **2e** (694 mg, 55.5%) was obtained as a brown waxy solid from 4-chloro-3-fluorobenzotrifluoride: ^{1}H NMR ($CDCl_3$, 500 MHz) δ 3.03-3.05 (m, 4H), 3.11-3.13 (m, 4H),

6.96 (t, *J* = 8.4 Hz, 1H), 7.25 (d, *J* = 12.8 Hz, 1H), 7.31 (d, *J* = 8.3 Hz, 1H); ^{13}C NMR ($CDCl_3$, 125 MHz) δ 155.4, 153.5, 143.2, 143.1, 121.5, 118.4, 118.3, 113.5, 113.4, 113.3, 113.2, 50.9, 45.7; LC-MS (ESI) *m*/*z:* 249.12 [M+H].

*1-(4-Fluoro-2-(2-fluoroethoxy)phenyl)piperazine (**2f**).* Following the general procedure, **2f** (593 mg, 38.7%) was obtained as a brown oil from 1-bromo-4-fluoro-2-(2-fluoroethoxy)benzene (**1f**): ^{1}H NMR ($CDCl_3$, 500 MHz) δ 3.00 (d, *J* = 4.6 Hz, 4H), 3.05 (t, *J* = 4.4 Hz, 4H), 4.18 (t, *J* = 4.0 Hz, 1H), 4.24 (t, *J* = 4.0 Hz, 1H), 4.71 (t, *J* = 4.0 Hz, 1H), 4.81 (t, *J* = 4.0 Hz, 1H), 6.58-6.65 (m, 2H), 6.84 (dd, *J* = 8.7, 6.0 Hz, 1H); ^{13}C NMR ($CDCl_3$, 125 MHz) δ 159.7, 157.8, 151.9, 151.8, 138.5, 119.0, 118.9, 107.5, 107.3, 101.8, 101.6, 82.3, 81.0, 67.8, 67.7, 52.0, 46.1; LC-MS (ESI) *m*/*z:* 243.25 [M+H].

*1-Bromo-4-fluoro-2-(2-fluoroethoxy)benzene (**1f**).* Compound 1f was produced according to the following procedure. 2-Bromo-5-fluorphenol (1 equiv), 1-fluoro-2-tosyloxyethane (1.1 equiv) and Cs_2CO_3 (4 equiv) solution in acetonitrile (35 mL) was stirred at 85 °C for 2 h, and the solvent was removed in vacuo. The reaction mixture was dissolved in water, extracted with EtOAc, and washed with brine. The organic layer was dried over Na_2SO_4 and concentrated. The crude mixture was purified by flash column chromatography on silica gel by elution with a 0–20 % gradient of EtOAc in hexane to give 1-bromo-4-fluoro-2- (2-fluoroethoxy)benzene (2.3 g, 91.4%) as a white solid: ^{1}H NMR ($CDCl_3$, 500 MHz) δ 4.19-4.20 (m, 1H), 4.24-4.26 (m, 1H), 4.73-4.74 (m, 1H), 4.82-4.84 (m, 1H), 6.58-6.65 (m, 2H), 7.47 (dd, *J* = 8.8, 6.2 Hz, 1H); ^{13}C NMR ($CDCl_3$, 125 MHz) δ 163.4, 161.5, 155.8, 155.7, 133.7, 133.6, 109.2, 109.0, 106.6, 106.5, 101.9, 101.7, 82.1, 80.7, 68.6, 68.4; LC-MS (ESI).

*General procedure for the synthesis of compound **3a–f**.* A solution of *N*-arylpiperazines **2a-f** (1 equiv), 1-bromobutane (10 equiv), and K_2CO_3 (20 equiv) in acetone was stirred at room temperature overnight. The crude mixture was filtered and concentrated under reduced pressure and purified by flash chromatography on silica gel by elution with a 0–16 % gradient of methanol in CH_2Cl_2. The products were converted to the corresponding hydrochloride salt.

*1-Butyl-4-(2-fluorophenyl)piperazine (**3a**).* Following the general procedure, **3a** (100 mg, 64.9%) was obtained as a light yellow oil: ^{1}H NMR ($CDCl_3$, 500 MHz) δ 0.92 (t, *J* = 7.4 Hz, 3H), 1.34 (dq, *J* = 15.0, 7.4 Hz, 2H), 1.47-1.53 (m, 2H), 2.39 (dd, *J* = 8.9, 7.7 Hz, 2H), 2.62 (s, 4H), 3.11 (t, *J* = 4.8 Hz, 4H), 6.88-6.95 (m, 2H), 6.97-7.05 (m, 2H); ^{13}C NMR ($CDCl_3$, 125 MHz) δ 156.6, 154.6, 140.1, 140.0, 124.3, 122.3, 122.2, 118.8, 116.0, 115.9, 58.4, 53.2, 50.4, 28.9, 20.7, 14.0; LC-MS (ESI) *m*/*z:* 237.25 [M+H].

*1-Butyl-4-(3-fluorophenyl)piperazine (**3b**).* Following the general procedure, **3b** (111 mg, 76.9%) was obtained as a light yellow oil: ^{1}H NMR ($CDCl_3$, 500 MHz) δ 0.94 (t, *J* = 7.4 Hz, 3H), 1.31-1.39 (m, 2H), 1.48-1.54 (m, 2H), 2.34 (t, *J* = 7.6 Hz, 2H), 2.57 (t, *J* = 4.7 Hz, 4H), 3.20 (t, *J* = 4.8 Hz, 4H), 6.50 (t, *J* = 8.1 Hz, 1H), 6.58 (d, *J* = 12.4 Hz, 1H), 6.66 (d, *J* = 8.3 Hz, 1H), 7.16 (q, *J* = 8.0 Hz, 1H); ^{13}C NMR ($CDCl_3$, 125 MHz) δ 164.7, 162.8, 153.0, 152.9, 130.0, 129.9, 110.9. 105.6, 105.4, 102.5, 102.3, 58.3, 53.0, 48.5, 29.0, 20.7, 14.0; LC-MS (ESI) *m*/*z:* 237.25 [M+H].

*1-Butyl-4-(4-fluorophenyl)piperazine (**3c**).* Following the general procedure, **3c** (110.2 mg, 70.0%) was obtained as a light yellow oil: ^{1}H NMR ($CDCl_3$, 500 MHz) δ 0.92 (t, *J* = 7.4 Hz, 3H), 1.33 (dq, *J* = 15.0, 7.4 Hz, 2H), 1.47-1.53 (m, 2H), 2.37 (dd, *J* = 9.8, 5.8 Hz, 2H), 2.58 (t, *J* = 5.0 Hz, 4H), 3.11 (t, *J* = 5.1 Hz, 4H), 6.83-6.87 (m, 2H), 6.90-6.96 (m, 2H); ^{13}C NMR ($CDCl_3$, 125 MHz) δ 157.9, 156.0, 147.9, 117.6, 117.5, 115.4, 115.2, 58.4, 53.2, 50.0, 28.9, 20.7, 14.0; LC-MS (ESI) *m*/*z:* 237.25 [M+H].

*3-(4-Butylpiperazin-1-yl)-4-fluorobenzonitrile (**3d**).* Following the general procedure, **3d** (46 mg, 63.4%) was obtained as a light yellow waxy solid: ^{1}H NMR ($CDCl_3$, 500 MHz) δ 0.92 (t, *J* = 7.4 Hz, 3H), 1.34 (dq, *J* = 15.0, 7.3 Hz, 2H), 1.46-1.51 (m, 2H), 2.39 (dd, *J* = 9.8, 7.6 Hz, 2H), 2.61 (t, *J* = 4.8 Hz, 4H), 3.13 (t, *J* = 10.0 Hz, 4H), 7.07 (dd, *J* = 12.2, 8.3 Hz, 1H), 7.16 (dd, *J* = 8.0, 2.0 Hz, 1H), 7.20-7.23 (m, 1H); ^{13}C NMR ($CDCl_3$, 125 MHz) δ 158.9, 156.9, 141.1, 141.0, 126.3, 122.5, 122.4, 118.5, 117.2, 117.0, 108.6, 58.3, 52.9, 50.0, 28.9, 20.6, 14.0; LC-MS (ESI) *m/z:* 262.19 [M+H].

*1-Butyl-4-(2-fluoro-4-(trifluoromethyl)phenyl)piperazine (**3e**).* Following the general procedure, **3e** (106.7 mg, 87.0%) was obtained as a yellow solid: ^{1}H NMR ($CDCl_3$, 500 MHz) δ 0.92 (t, *J* = 7.4 Hz, 3H), 1.34 (td, *J* = 14.8, 7.4 Hz, 2H), 1.47-1.53 (m, 2H), 2.38 (dd, *J* = 9.8, 7.6 Hz, 2H), 2.60 (t, *J* = 4.9 Hz, 4H), 3.17 (t, *J* = 5.0 Hz, 4H), 6.95 (t, *J* = 8.4 Hz, 1H), 7.23 (dd, *J* = 12.8, 1.9 Hz, 1H), 7.29 (dd, *J* = 8.4, 0.6 Hz, 1H); ^{13}C NMR ($CDCl_3$, 125 MHz) δ 155.5, 153.6, 143.1, 143.0, 121.7, 118.4, 113.6, 113.4, 58.4, 53.0, 50.0, 49.9, 29.0, 20.7, 13.9; LC-MS (ESI) *m/z:* 305.15 [M+H].

*1-Butyl-4-(4-fluoro-2-(2-fluoroethoxy)phenyl)piperazine (**3f**).* Following the general procedure, **3f** (39 mg, 70.4%) was obtained as a brown oil: ^{1}H NMR ($CDCl_3$, 500 MHz) δ 0.92 (t, *J* = 7.4 Hz, 3H), 1.30-1.38 (m, 2H), 1.49-1.55 (m, 2H), 2.42 (dd, *J* = 9.8, 7.7 Hz, 2H), 2.65 (br, 4H), 3.08 (br, 1H), 4.17-4.19 (m, 1H), 4.23-4.24 (m, 1H), 4.71-7.72 (m, 1H), 4.80-4.82 (m, 1H), 6.58 (dd, *J* = 10.0, 2.8 Hz, 1H), 6.63 (td, *J* = 11.0. 8.2, 2.8 Hz, 1H), 6.85 (dd, *J* = 8.8, 6.0 Hz, 1H); ^{13}C NMR ($CDCl_3$, 125 MHz) δ 159.7, 157.8, 151.8, 151.7, 138.2, 138.1, 118.8, 118.7, 107.5, 107.3, 101.8, 101.6, 82.3, 81.0, 67.8, 67.6, 58.5, 53.4, 50.7, 28.8, 20.7, 14.0; LC-MS (ESI) *m/z:* 299.28 [M+H].

*General procedure for the synthesis of compound **4a–f**.* A solution of *N*-arylpiperazines **2a-f** (1 equiv), *N*-(4-bromobutyl)phthlimide (3 equiv), and Et_3N (10 equiv) in acetonitrile was stirred at room temperature overnight and the solvent was removed in vacuo. The reaction mixture was dissolved in CH_2Cl_2 and washed with saturated $NaHCO_3$ solution and brine. The organic layer was dried over Na_2SO_4 and concentrated under reduced pressure. The crude mixture was purified by flash chromatography on silica gel by elution with a 0–16 % gradient of methanol in CH_2Cl_2 to give the target compound.

*2-(4-(4-(2-Fluorophenyl)piperazin-1-yl)butyl)isoindoline-1,3-dione (**4a**).* Following the general procedure, **4a** (591 mg, 94.7%) was obtained as a light brown solid: ^{1}H NMR ($CDCl_3$, 500 MHz) δ 1.55-1.61 (m, 2H), 1.71-1.77 (m, 2H), 2.45 (t, *J* = 7.4 Hz, 2H), 2.63 (br, 4H), 3.10 (br, 4H), 3.72 (t, *J* = 7.2 Hz, 2H), 6.90-6.95 (m, 2H), 6.98-7.06 (m, 2H), 7.69-7.73 (m, 2H), 7.81-7.86 (m, 2H); ^{13}C NMR ($CDCl_3$, 125 MHz) δ 168.4, 156.6, 154.7, 140.1, 140.0, 133.9, 132.1, 124.4, 123.1, 122.4, 122.3, 118.9, 118.8, 116.1, 115.9, 58.0, 53.2, 50.4, 37.8, 26.5, 24.1; LC-MS (ESI) *m/z:* 382.16 [M+H].

*2-(4-(4-(3-Fluorophenyl)piperazin-1-yl)butyl)isoindoline-1,3-dione (**4b**).* Following the general procedure, **4b** (814 mg, 91.2%) was obtained as a yellow solid: ^{1}H NMR ($CDCl_3$, 500 MHz) δ 1.47-1.54 (m, 2H), 1.65-1.71 (m, 2H), 2.35 (t, *J* = 7.6 Hz, 2H), 2.49 (t, *J* = 5.0 Hz, 4H), 3.10 (t, *J* = 5.0 Hz, 4H), 3.66 (t, *J* = 7.1 Hz, 2H), 6.42 (td, *J* = 8.2, 2.2 Hz, 1H), 6.49 (dt, *J* = 12.4, 4.4 Hz, 1H), 6.57 (dd, *J* = 8.3, 2.0 Hz, 1H), 7.08 (q, *J* = 8.2 Hz, 1H), 7.61-7.64 (m, 2H), 7.73-7.77 (m, 2H); ^{13}C NMR ($CDCl_3$, 125 MHz) δ 168.0, 164.5, 162.6, 152.7, 152.6, 133.6, 131.8, 129.8, 129.7, 122.8, 110.7, 105.4, 105.2, 102.3, 102.1, 57.6, 52.7, 48.2, 37.5, 26.3, 23.9; LC-MS (ESI) *m/z:* 382.16 [M+H].

*2-(4-(4-(4-Fluorophenyl)piperazin-1-yl)butyl)isoindoline-1,3-dione (**4c**).* Following the general procedure, **4c** (378 mg, 74.6%) was obtained as a yellow solid: ^{1}H NMR ($CDCl_3$, 500 MHz) δ 1.60 (br, 4H), 1.71-1.77 (m, 2H), 2.46 (br, 1H), 2.63 (br, 3H), 3.13 (br, 4H), 3.73 (t, *J* = 7.1 Hz, 2H), 6.85-6.88 (m, 2H), 6.93-6.97 (m, 2H), 7.70-7.73 (m, 2H), 7.82-7.85 (m, 2H); ^{13}C NMR

($CDCl_3$, 125 MHz) δ 168.4, 133.9, 132.1, 123.2, 117.8, 117.7, 115.6, 115.4, 57.9, 53.2, 50.1, 37.8, 26.6, 24.1; LC-MS (ESI) *m/z:* 382.16 [M+H].

*3-(4-(4-(1,3-Dioxoisoindolin-2-yl)butyl)piperazin-1-yl)-4-fluorobenzonitrile (**4d**).* Following the general procedure, **4d** (522 mg, 71.2%) was obtained as a brown solid: ^{1}H NMR ($CDCl_3$, 500 MHz) δ 1.60 (br, 5H), 1.72-1.78 (m, 2H), 2.49-2.66 (m, 5H), 3.15 (br, 2H), 3.71-3.74 (m, 2H), 6.84-7.25 (m, 3H), 7.70-7.74 (m, 2H), 7.81-7.86 (m, 2H); ^{13}C NMR ($CDCl_3$, 125 MHz) δ 168.4, 133.9, 133.4, 132.1, 129.8, 129.7, 126.5, 126.4, 123.2, 122.6, 122.5, 118.5, 117.3, 117.1, 115.6, 114.1, 108.7, 57.8, 52.9, 52.7, 49.9, 47.0, 37.7, 26.5, 24.0; LC-MS (ESI) *m/z:* 407.25 [M+H].

*2-(4-(4-(2-Fluoro-4-(trifluoromethyl)phenyl)piperazin-1-yl)butyl)isoindoline-1,3-dione (**4e**).* Following the general procedure, **4e** (605 mg, 86.8%) was obtained as a yellow solid: ^{1}H NMR ($CDCl_3$, 500 MHz) δ 1.53-1.58 (m, 2H), 1.71-1.77 (m, 2H), 2.43 (t, *J* = 7.6 Hz, 2H), 2.60 (t, *J* = 4.8 Hz, 4H), 3.16 (t, *J* = 5.0 Hz, 4H), 3.72 (t, *J* = 7.2 Hz, 2H), 6.94 (t, *J* = 8.4 Hz, 1H), 7.24 (dd, *J* = 12.9, 2.0 Hz, 1H), 7.30 (d, *J* = 8.4 Hz, 1H), 7.70 (dd, *J* = 5.4, 3.0 Hz, 2H), 7.83 (dd, *J* = 5.4, 3.0 Hz, 2H); ^{13}C NMR ($CDCl_3$, 125 MHz) δ 168.4, 155.6, 153.6, 133.9, 132.1, 123.2, 121.7, 118.5, 118.4, 113.6, 113.4, 57.9, 53.0, 50.0, 49.9, 37.8, 26.5, 24.1; LC-MS (ESI) *m/z:* 450.11 [M+H].

*2-(4-(4-(4-Fluoro-2-(2-fluoroethoxy)phenyl)piperazin-1-yl)butyl)isoindoline-1,3-dione (**4f**).* Following the general procedure, **4f** (781 mg, 77.9%) was obtained as a brown oil: ^{1}H NMR ($CDCl_3$, 500 MHz) δ 1.51-1.57 (m, 2H), 1.67-1.73 (m, 2H), 2.40 (t, *J* = 7.5 Hz, 2H), 2.58 (s, 4H), 3.00 (s, 4H), 3.68 (t, *J* = 7.2 Hz, 2H), 4.15 (t, *J* = 4.1 Hz, 1H), 4.20 (t, *J* = 4.0 Hz, 1H), 4.67-4.69 (m, 1H), 4.77-4.78 (m, 1H), 6.54-6.61 (m, 1H), 6.80 (dd, *J* = 8.8, 6.0 Hz, 1H), 7.65-7.68 (m, 2H), 7.77-7.80 (m, 2H); ^{13}C NMR ($CDCl_3$, 125 MHz) δ 168.2, 159.5, 157.6, 151.7, 151.6, 138.1, 133.7, 132.0, 123.0, 118.6, 118.5, 107.3, 107.2, 101.6, 101.4, 82.2, 80.9, 67.7, 67.5, 57.9, 53.3, 50.7, 37.7, 26.4, 24.0; LC-MS (ESI) *m/z:* 444.10 [M+H].

*General procedure for the synthesis of compound **5a–f**.* Hydrazine hydrate (5.5 equiv) was added to a solution of phthalimide compounds **4a-f** (1 equiv) in ethanol, and the reaction mixture was refluxed for 30 min. After cooling to room temperature, the crude mixture was filtered, and concentrated under reduced pressure. The crude product was purified by flash chromatography on silica gel by elution with 0–20 % gradient of 7N ammonia solution in methanol and CH_2Cl_2 to give the target compound.

*4-(4-(2-Fluorophenyl)piperazin-1-yl)butan-1-amine (**5a**).* Following the general procedure, **5a** (84 mg, 86.8%) was obtained as a light yellow oil: ^{1}H NMR ($CDCl_3$, 500 MHz) δ 1.43-1.55 (m, 4H), 2.32-2.37 (m, 4H), 2.58 (s, 4H), 2.68 (t, *J* = 6.8 Hz, 2H), 3.06 (t, *J* = 4.2 Hz, 4H) 6.84-6.91 (m, 2H), 6.93-7.01 (m, 2H); ^{13}C NMR ($CDCl_3$, 125 MHz) δ 156.5, 154.6, 140.0, 139.9, 124.3, 124.2, 122.2, 118.7. 116.0, 115.8, 58.3, 53.1, 50.3, 41.7, 31.1, 24.1; LC-MS (ESI) *m/z:* 252.32 [M+H].

*4-(4-(3-Fluorophenyl)piperazin-1-yl)butan-1-amine (**5b**).* Following the general procedure, **5b** (322 mg, 78.9 %) was obtained as a light yellow oil; ^{1}H NMR ($CDCl_3$, 500 MHz) δ 1.29-1.34 (m, 4H), 1.36-1.42 (m, 2H), 2.22 (t, *J* = 7.6 Hz, 2H), 2.40 (t, *J* = 5.0 Hz, 4H), 2.55 (t, *J* = 5.8 Hz, 2H), 3.03 (t, *J* = 5.1 Hz, 4H),6.35 (td, *J* = 8.2, 2.0 Hz, 1H), 6.42 (dt, *J* = 12.5, 2.2 Hz, 1H), 6.50 (dd, *J* = 8.4, 2.0 Hz, 1H), 7.01 (q, *J* = 8.2 Hz, 1H); ^{13}C NMR ($CDCl_3$, 125 MHz) δ 164.3, 162.4, 152.6, 152.5, 129.6, 129.5, 110.5, 105.1, 105.0, 102.0, 101.8, 57.9, 52.5, 48.0, 41.6, 31.2, 23.8; LC-MS (ESI) *m/z:* 252.18 [M+H].

*4-(4-(4-Fluorophenyl)piperazin-1-yl)butan-1-amine (**5c**).* Following the general procedure, **5c** (69 mg, 94.9%) was obtained as a light yellow oil: ^{1}H NMR ($CDCl_3$, 500 MHz) δ 1.49-1.56 (m, *J* = 6.0 Hz, 4H), 2.39 (t, *J* = 7.6 Hz, 4H), 2.59-2.60 (t, *J* = 4.3 Hz, 4H), 2.73 (t, *J* = 6.0 Hz, 2H), 3.11 (t, *J* = 4.2 Hz, 4H), 6.85 (dd, *J* = 8.6, 4.4 Hz, 2H), 6.93 (t, *J* = 8.6 Hz, 2H); ^{13}C NMR

($CDCl_3$, 125 MHz) δ 158.0, 156.1, 147.9, 117.8, 117.7, 115.5, 115.3, 58.3, 53.1, 50.0, 41.8, 31.2, 29.6, 24.3; LC-MS (ESI) *m/z:* 252.18 [M+H].

*3-(4-(4-Aminobutyl)piperazin-1-yl)-4-fluorobenzonitrile (**5d**).* Following the general procedure, **5d** (151 mg, 69.6%) was obtained as a light yellow oil: ^{1}H NMR ($CDCl_3$, 500 MHz) δ 1.48-1.54 (m, 4H), 2.34-2.38 (m, 2H), 2.51-2.53 (m, 2H), 2.57 (t, *J* = 4.4 Hz, 4H), 2.70 (br, 2H), 3.08 (t, *J* = 4.6 Hz, 4H), 7.03 (dd, *J* = 12.2, 8.4 Hz, 1H), 7.13 (dd, *J* = 8.0, 1.1 Hz, 1H), 7.17-7.20 (m, 1H); ^{13}C NMR ($CDCl_3$, 125 MHz) δ 158.8, 156.8, 153.2, 141.0, 133.3, 126.3, 122.4, 119.9, 118.4, 117.2, 117.0, 114.0, 108.5, 100.0, 58.1, 53.1, 52.8, 52.6, 49.9, 46.9, 41.6, 31.0, 29.5, 24.1; LC-MS (ESI) *m/z:* 277.40 [M+H].

*4-(4-(2-Fluoro-4-(trifluoromethyl)phenyl)piperazin-1-yl)butan-1-amine (**5e**).* Following the general procedure, **5e** (234 mg, 83.2%) was obtained as a light yellow oil: ^{1}H NMR ($CDCl_3$, 500 MHz) δ 1.36-1.48 (m, 4H), 1.84 (s, 2H), 2.30 (t, *J* = 7.6 Hz, 2H), 2.50 (t, *J* = 4.8 Hz, 4H), 2.62 (t, *J* = 6.6 Hz, 2H), 3.06 (t, *J* = 4.9 Hz, 4H), 6.84 (t, *J* = 8.4 Hz, 1H), 7.13 (dd, *J* = 12.8, 1.8 Hz, 1H), 7.18 (d, *J* = 8.4 Hz, 1H); ^{13}C NMR ($CDCl_3$, 125 MHz) δ 155.3, 153.3, 142.8, 142.7, 121.5, 121.4, 118.2, 113.3, 113.1, 58.1, 52.8, 49.7, 49.6, 41.7, 31.1, 24.0; LC-MS (ESI) *m/z:* 320.36 [M+H].

*4-(4-(4-Fluoro-2-(2-fluoroethoxy)phenyl)piperazin-1-yl)butan-1-amine (**5f**).* Following the general procedure, **5f** (370 mg, 67.2%) was obtained as a light yellow oil: ^{1}H NMR ($CDCl_3$, 500 MHz) δ 1.32-1.38 (m, 2H), 1.39-1.45 (m, 2H), 1.59 (br, 2H), 2.26 (t, *J* = 7.2 Hz, 2H), 2.58 (t, *J* = 6.8 Hz, 2H), 2.91 (br, 1H), 4.03 (t, *J* = 4.1 Hz, 1H), 4.09 (t, *J* = 4.1 Hz, 1H), 4.57 (t, *J* = 4.1 Hz, 1H), 4.66 (t, *J* = 4.1 Hz, 1H), 6.45 (dd, *J* = 10.1, 2.8 Hz, 1H), 6.49 (td, *J* = 11.2, 8.4, 2.8 Hz, 1H), 6.71 (dd, *J* = 8.8, 6.0 Hz, 1H); ^{13}C NMR ($CDCl_3$, 125 MHz) δ 159.2, 157.3, 151.5, 151.4, 137.9, 118.4, 118.3, 107.1, 106.9, 101.4, 101.2, 82.0, 80.6, 67.5, 67.3, 58.1, 53.1, 50.5, 41.6, 31.2, 23.9; LC-MS (ESI) *m/z:* 314.22 [M+H].

*General procedure for the synthesis of compound **6a–f** and **7a–f**.* Primary amines **5a-f** (1 equiv), 4-(thien-3-yl)benzoic acid (0.8 equiv) or 4-(1,3-thiazol-4-yl)benzoic acid (0.8 equiv) were dissolved in CH_2Cl_2. To the solution, EDC (1.2 equiv) and HOBt hydrate (1.2 equiv) was added at 0 °C and stirred at room temperature for 1 h. The crude mixture was washed with saturated $NaHCO_3$ solution and brine. The organic layer was dried over Na_2SO_4 and concentrated. The product was purified by flash column chromatography on silica gel by elution with a 0–10 % gradient of methanol in CH_2Cl_2 and converted to the corresponding hydrochloride salt.

*N-(4-(4-(2-Fluorophenyl)piperazin-1-yl)butyl)-4-(thiophen-3-yl)benzamide (**6a**).* Following the general procedure, **6a** (100 mg, 74.9%) was obtained as a white solid: ^{1}H NMR ($CDCl_3$, 500 MHz) δ 1.66-1.73 (m, 4H), 2.48 (t, *J* = 10.0 Hz, 2H), 2.64 (s, 4H), 3.09 (s, 4H), 3.50 (dd, *J* = 10.0, 5.0 Hz, 2H), 6.74 (br, 1H), 6.87-6.94 (m, 2H), 6.98-7.02 (m, 2H), 7.39-7.41 (m, 2H), 7.50-7.51 (m, 1H), 7.63 (d, *J* = 10.0 Hz, 2H), 7.80 (d, *J* = 5.0 Hz, 2H); ^{13}C NMR ($CDCl_3$, 125 MHz) δ 167.3, 156.7, 154.7, 141.2, 140.0, 138.6, 133.4, 127.6, 126.6, 126.4, 126.1, 124.4, 122.5, 121.4, 118.9, 116.2, 116.0, 58.0, 53.2, 50.3, 50.2, 39.9, 27.4, 24.3; LC-MS (ESI) *m/z:* 438.23 [M+H].

*N-(4-(4-(3-Fluorophenyl)piperazin-1-yl)butyl)-4-(thiophen-3-yl)benzamide (**6b**).* Following the general procedure, **6b** (172 mg, 85.1%) was obtained as a white solid: ^{1}H NMR ($CDCl_3$, 500 MHz) δ 1.64-1.72 (m, 4H), 2.45 (t, *J* = 6.8 Hz, 2H), 2.58 (t, *J* = 4.9 Hz, 4H), 3.17 (t, *J* = 5.0 Hz, 4H), 3.50 (q, *J* = 6.2 Hz, 2H), 6.49-6.57 (m, 2H), 6.62-6.66 (m, 2H), 7.16 (q, *J* = 8.2 Hz, 1H), 7.38-7.41 (m, 2H), 7.50 (q, *J* = 1.4 Hz, 1H), 7.62 (d, *J* = 8.2 Hz, 2H), 7.78 (d, *J* = 8.3 Hz, 2H); ^{13}C NMR ($CDCl_3$, 125 MHz) δ 167.3, 164.8, 162.8, 152.8, 141.1, 138.6, 133.3, 130.1, 130.0, 127.5, 126.6, 126.3, 126.1, 121.4, 111.0, 105.9, 105.7, 102.7, 102.5, 57.9, 53.0, 48.4, 39.9, 27.4, 24.3; LC-MS (ESI) *m/z:* 438.10 [M+H].

N-(4-(4-(4-Fluorophenyl)piperazin-1-yl)butyl)-4-(thiophen-3-yl)benzamide (***6c***). Following the general procedure, **6c** (62 mg, 52.0%) was obtained as a white solid: ^{1}H NMR ($CDCl_3$, 500 MHz) δ 1.65-1.72 (m, 4H), 2.46 (t, *J* = 6.8 Hz, 2H), 2.61 (t, *J* = 4.9 Hz, 4H), 3.09 (t, *J* = 5.0 Hz, 4H), 3.40 (dd, *J* = 12.1, 6.4 Hz, 2H), 6.82-6.85 (m, 2H), 6.91-6.95 (m, 2H), 7.38-7.42 (m, 2H), 7.50-7.51 (m, 1H), 7.62 (dd, *J* = 6.6, 1.8 Hz, 2H), 7.79 (d, *J* = 8.4 Hz, 2H); ^{13}C NMR ($CDCl_3$, 125 MHz) δ 167.3, 156.2, 147.8, 141.1, 138.5, 133.3, 127.5, 126.6, 126.3, 126.1, 121.4, 117.8, 117.7, 115.5, 115.4, 57.9, 53.1, 49.9, 39.9, 29.6, 27.4, 24.3; LC-MS (ESI) *m*/*z:* 438.23 [M+H].

N-(4-(4-(5-Cyano-2-fluorophenyl)piperazin-1-yl)butyl)-4-(thiophen-3-yl)benzamide (***6d***). Following the general procedure, **6d** (99 mg, 88.3%) was obtained as a light yellow solid: ^{1}H NMR ($CDCl_3$, 500 MHz) δ 1.62-1.71 (m, 4H), 2.41-2.47 (m, 2H), 2.54 (t, *J* = 5.0 Hz, 1H), 2.60 (t, *J* = 4.6 Hz, 3H), 3.09 (t, *J* = 4.8 Hz, 3H), 3.26 (t, *J* = 5.2 Hz, 1H), 3.49 (dd, *J* = 12.4, 6.6 Hz, 2H), 6.77-6.78 (m, 1H), 7.06 (dd, *J* = 12.2, 8.4 Hz, 1H), 7.11 (dd, *J* = 8.0, 2.0 Hz, 1H), 7.21 (ddd, *J* = 6.2, 4.2, 2.0 Hz, 1H), 7.37-7.40 (m, 2H), 7.48-7.50 (m, 1H), 7.60-7.63 (m, 2H), 7.77-7.80 (m, 2H); ^{13}C NMR ($CDCl_3$, 125 MHz) δ 167.2, 158.9, 156.9, 153.2, 141.1, 141.0, 140.9, 138.6, 133.4, 133.2, 127.5, 126.6, 126.5, 126.4, 126.3, 126.0, 122.4, 121.4, 120.0, 118.4, 117.3, 117.1, 114.0, 108.6, 108.6, 57.9, 52.9, 52.6, 49.8, 46.9, 39.9, 27.4, 24.2; LC-MS (ESI) *m*/*z:* 463.20 [M+H].

N-(4-(4-(2-Fluoro-4-(trifluoromethyl)phenyl)piperazin-1-yl)butyl)-4-(thiophen-3-yl)benzamide (***6e***). Following the general procedure, **6e** (97 mg, 73.1%) was obtained as a white solid: ^{1}H NMR ($CDCl_3$, 500 MHz) δ 1.72 (br, 4H), 2.53 (s, 2H), 2.68 (s, 4H), 3.18 (s, 4H), 3.52 (d, *J* = 2.7 Hz, 2H), 6.66 (br, 1H), 6.91 (t, *J* = 8.1 Hz, 1H), 7.24-7.28 (m, 2H), 7.40 (br, 2H), 7.52 (s, 1H), 7.64 (d, *J* = 7.8 Hz, 2H), 7.80 (d, *J* = 7.6 Hz, 2H); ^{13}C NMR ($CDCl_3$, 125 MHz) δ 167.3, 157.9, 153.6, 151.7, 141.1, 138.4, 137.6, 133.3, 127.5, 126.6, 126.4, 126.1, 121.7, 121.4, 118.5, 113.5, 57.9, 53.0, 49.7, 47.7, 39.6, 29.7, 28.5, 27.4; LC-MS (ESI) *m*/*z:* 506.07 [M+H].

N-(4-(4-(2-(2-Fluoroethoxy)-4-(trifluoromethyl)phenyl)piperazin-1-yl)butyl)-4-(thiophen-3-yl)benzamide (***6f***). Following the general procedure, **6f** (52 mg, 75.2%) was obtained as a yellow solid: ^{1}H NMR ($CDCl_3$, 500 MHz) δ 1.70 (s, 4H), 2.54 (t, *J* = 6.4 Hz, 2H), 2.71 (br, 4H), 3.06 (br, 4H), 3.48 (d, *J* = 2.8 Hz, 2H), 4.16 (t, *J* = 4.0 Hz, 1H), 4.22 (t, *J* = 4.0 Hz, 1H), 4.70 (t, *J* = 4.0 Hz, 1H), 4.79 (t, *J* = 4.0 Hz, 1H), 6.56-6.60 (m, 2H), 6.77 (dd, *J* = 9.4, 6.8 Hz, 1H), 7.03 (br, 1H), 7.38 (dd, *J* = 7.6, 5.0 Hz, 2H), 7.49 (dd, *J* = 2.4, 1.6 Hz, 1H), 7.62 (d, *J* = 8.3 Hz, 2H), 7.82 (d, *J* = 8.4 Hz, 2H); ^{13}C NMR ($CDCl_3$, 125 MHz) δ 167.3, 159.8, 157.9, 151.8, 151.7, 141.1, 138.4, 137.6, 133.3, 127.6, 126.5, 126.2, 126.1, 121.3, 118.9, 118.8, 107.5, 107.3, 101.7, 101.5, 82.3, 80.9, 67.8, 67.6, 57.7, 53.2, 50.1, 39.6, 27.2, 23.7; LC-MS (ESI) *m*/*z:* 499.93 [M+H].

N-(4-(4-(2-Fluorophenyl)piperazin-1-yl)butyl)-4-(thiazol-4-yl)benzamide (***7a***). Following the general procedure, **7a** (78 mg, 75.6%) was obtained as a white solid: ^{1}H NMR ($CDCl_3$, 500 MHz) δ 1.64-1.70 (m, 4H), 2.47 (t, *J* = 6.8 Hz, 2H), 2.64 (s, 4H), 3.08 (t, *J* = 4.6 Hz, 4H), 3.48 (dd, *J* = 12.0, 6.2 Hz, 2H), 6.85-6.92 (m, 2H), 6.94-7.00 (m, 3H), 7.58 (d, *J* = 2.0 Hz, 1H), 7.84 (d, *J* = 8.4 Hz, 2H), 7.96 (d, *J* = 8.4 Hz, 2H), 8.86 (d, *J* = 1.9 Hz, 1H); ^{13}C NMR ($CDCl_3$, 125 MHz) δ 167.2, 156.6, 155.2, 154.6, 153.0, 139.8, 136.8, 134.3, 127.6, 126.4, 124.4, 122.5, 122.4, 118.9, 116.1, 115.9, 114.0, 57.8, 53.1, 50.1, 39.8, 27.3, 24.1; LC-MS (ESI) *m*/*z:* 439.30 [M+H].

N-(4-(4-(3-Fluorophenyl)piperazin-1-yl)butyl)-4-(thiazol-4-yl)benzamide (***7b***). Following the general procedure, **7b** (127 mg, 83.5%) was obtained as a white solid: ^{1}H NMR ($CDCl_3$, 500 MHz) δ 1.64-1.72 (m, 4H), 2.45 (t, *J* = 6.8 Hz, 2H), 2.58 (t, *J* = 4.8 Hz, 4H), 3.16 (t, *J* = 5.0 Hz, 4H), 3.49 (q, *J* = 6.2 Hz, 2H), 6.48-6.55 (m, 2H), 6.62 (dd, *J* = 8.3, 1.8 Hz, 1H), 6.69 (br, 1H), 7.14 (q, *J* = 8.0 Hz, 1H), 7.59 (d, *J* = 1.8 Hz, 1H), 7.82 (d, *J* = 8.2 Hz, 2H), 7.97 (d, *J* = 8.2 Hz, 2H), 8.88 (d, *J* = 1.8 Hz, 1H); ^{13}C NMR ($CDCl_3$, 125 MHz) δ 167.2, 164.8, 162.8, 155.2, 153.1, 152.8, 152.7, 136.9, 134.4, 130.1, 130.0, 127.5, 126.4, 114.0, 111.1, 105.9, 105.7, 102.7, 102.5, 57.9, 53.0, 48.4, 39.9, 27.4, 24.3; LC-MS (ESI) *m*/*z:* 439.03 [M+H].

N-(4-(4-(4-Fluorophenyl)piperazin-1-yl)butyl)-4-(thiazol-4-yl)benzamide (***7c***). Following the general procedure, **7c** (40 mg, 65.2%) was obtained as a white solid: ^{1}H NMR ($CDCl_3$, 500 MHz) δ 1.70 (br, 4H), 2.48 (br, 2H), 2.62 (s, 4H), 3.09 (s, 4H), 3.50 (d, *J* = 5.4 Hz, 2H), 6.77-6.83 (m, 3H), 6.92 (t, *J* = 8.2 Hz, 2H), 7.60 (s, 1H), 7.83 (d, *J* = 7.6 Hz, 2H), 7.97 (d, *J* = 7.8 Hz, 2H), 8.89 (s, 1H); ^{13}C NMR ($CDCl_3$, 125 MHz) δ 167.3, 155.2, 153.1, 147.7, 136.8, 134.4, 127.5, 126.4, 117.9, 117.8, 115.6, 115.4, 114.0, 57.9, 53.1, 49.9, 39.9, 29.7, 27.4, 24.2; LC-MS (ESI) *m/z:* 439.03 [M+H].

N-(4-(4-(5-Cyano-2-fluorophenyl)piperazin-1-yl)butyl)-4-(thiazol-4-yl)benzamide (***7d***). Following the general procedure, **7d** (84 mg, 68.5%) was obtained as a yellow solid: ^{1}H NMR ($CDCl_3$, 500 MHz) δ 1.62-1.68 (m, 4H), 2.42-2.43 (m, 2H), 2.52-2.58 (m, 4H), 3.06 (s, 3H), 3.24 (s, 1H), 3.47 (d, *J* = 5.2 Hz, 2H), 6.76-6.83 (m, 1H), 7.02-7.09 (m, 2H), 7.18 (s, 1H), 7.56 (d, *J* = 11.8 Hz, 2H), 7.95 (t, *J* = 10.3 Hz, 2H), 8.86 (s, 1H); ^{13}C NMR ($CDCl_3$, 125 MHz) δ 167.2, 158.8, 156.8, 155.1, 153.1, 140.9, 140.8, 136.8, 134.3, 133.3, 127.5, 126.3, 127.4, 118.4, 117.2, 117.0, 114.0, 113.7, 108.6, 57.8, 52.8, 52.6, 49.7, 46.8, 39.9, 27.4, 24.2; LC-MS (ESI) *m/z:* 464.40 [M+H].

N-(4-(4-(2-Fluoro-4-(trifluoromethyl)phenyl)piperazin-1-yl)butyl)-4-(thiazol-4-yl)benzamide (***7e***). Following the general procedure, **7e** (109 mg, 75.0%) was obtained as a white solid. ^{1}H NMR ($CDCl_3$, 500 MHz) δ 1.65-1.74 (m, 4H), 2.48 (t, *J* = 6.9 Hz, 2H), 2.62 (t, *J* = 4.7 Hz, 4H), 3.14 (t, *J* = 4.9 Hz, 4H), 3.50 (dd, *J* = 12.2, 5.4 Hz, 2H), 6.70 (br, 1H), 6.89 (t, *J* = 8.4 Hz, 1H), 7.25 (t, *J* = 11.0 Hz, 2H) 7.61 (d, *J* = 2.0 Hz, 1H), 7.83 (d, *J* = 8.4 Hz, 2H), 7.99 (dd, *J* = 6.6, 1.6 Hz, 2H), 8.89 (d, *J* = 1.9 Hz, 1H); ^{13}C NMR ($CDCl_3$, 125 MHz) δ 167.3, 155.6, 155.3, 153.6, 153.1, 136.9, 134.4, 127.5, 126.5, 121.7, 118.5, 114.0, 113.6, 113.4, 58.0, 53.0, 49.8, 49.7, 40.0, 27.5, 24.3; LC-MS (ESI) *m/z:* 507.00 [M+H].

N-(4-(4-(4-Fluoro-2-(2-fluoroethoxy)phenyl)piperazin-1-yl)butyl)-4-(thiazol-4-yl)benzamide (***7f***). Following the general procedure, **7f** (61 mg, 63.7%) was obtained as a yellow solid: ^{1}H NMR ($CDCl_3$, 500 MHz) δ 1.69 (s, 4H), 2.54 (t, *J* = 6.6 Hz, 2H), 2.72 (br, 4H), 3.06 (br, 4H), 3.47 (d, *J* = 5.6 Hz, 2H), 4.14 (t, *J* = 4.0 Hz, 1H), 4.20 (t, *J* = 4.0 Hz, 1H), 4.68 (dd, *J* = 5.5, 4.0 Hz, 1H), 4.78 (dd, *J* = 5.5, 4.0 Hz, 1H), 6.54-6.58 (m, 2H), 6.76 (dd, *J* = 9.4, 6.2 Hz, 1H), 7.12 (br, 1H), 7.59 (d, *J* = 1.9 Hz, 1H), 7.86 (d, *J* = 8.4 Hz, 2H), 7.95 (d, *J* = 8.4 Hz, 2H), 8.86 (d, (d, *J* = 1.9 Hz, 1H); ^{13}C NMR ($CDCl_3$, 125 MHz) δ 167.2, 159.8, 157.8, 155.2, 153.0, 151.8, 151.7, 137.6, 137.5, 136.7, 134.3, 127.6, 126.3, 118.9, 118.8, 113.9, 107.4, 107.2, 101.7, 101.5, 82.2, 80.9, 67.8, 67.6, 57.7, 53.4, 53.1, 50.0, 39.6, 27.1, 23.6; LC-MS (ESI) *m/z:* 501.13 [M+H].

2. Comparison of Competitive Radioligand Binding Experiments Using D2L and D3 Dopamine Receptors

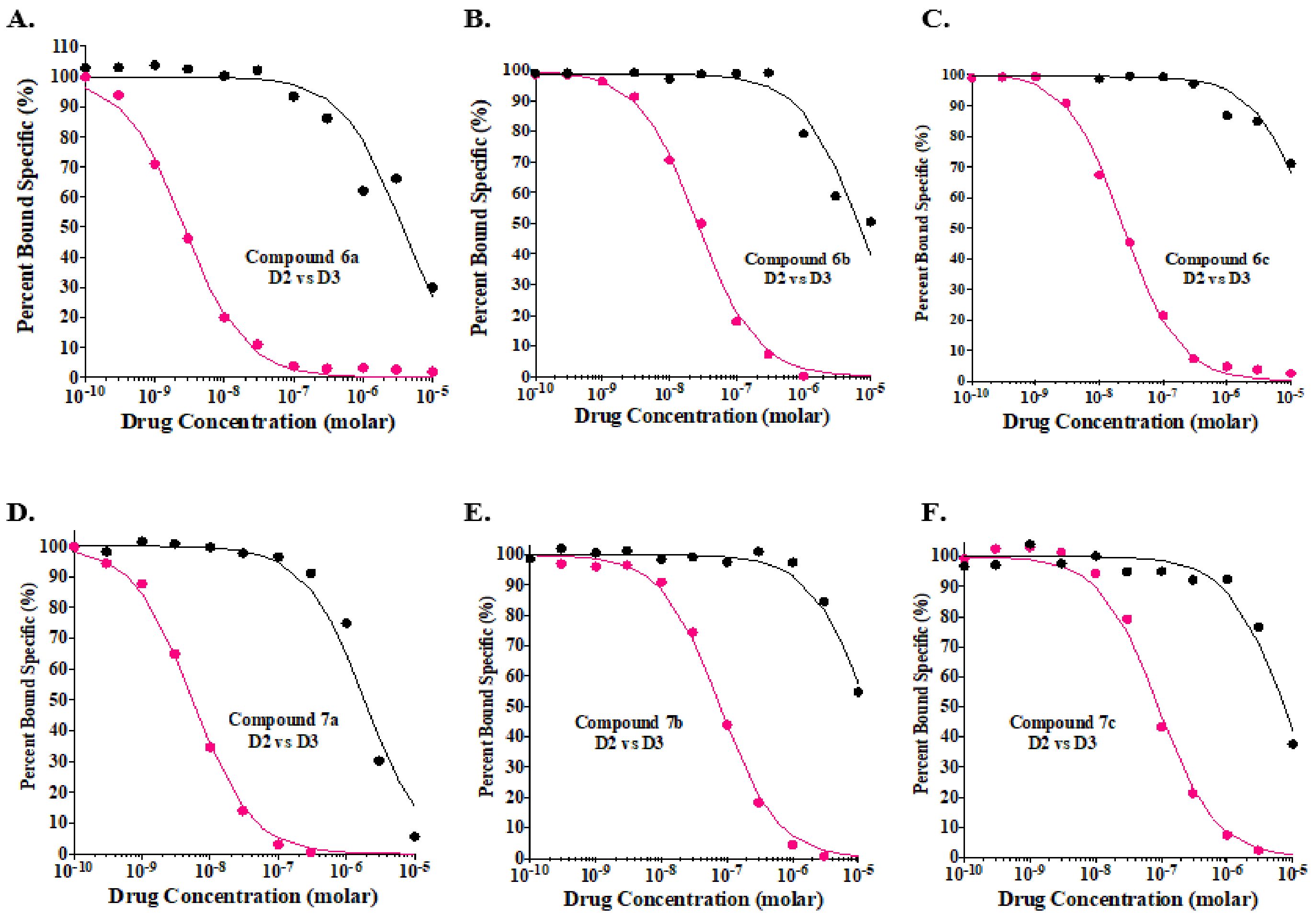

Figure S1. Representative examples of competitive radioligand binding curves are shown for A) compound **6a**, B) compound **6b**, C) compound **6c**, D) compound **7a**, E) compound **7b** and F) compound **7c** at human D2L (-●-) and D3 (-●-) dopamine receptors expressed in stably transfected HEK cells using the radioligand ^{125}I-IABN are shown. The Ki values for binding of these compounds at the human D2L and D3 dopamine receptors, as well as the structure of these fluorine substituted thiophene (compounds **6a-c**) and thiazolylphenyl (compounds **7a-c**) phenylpiperazines compounds are shown in **Table 1**.
